# RUNX1-regulated ITGB1-enriched extracellular vesicles drive pancreatic cancer liver metastasis via fibrotic pre-metastatic niche formation

**DOI:** 10.1186/s12967-026-08521-3

**Published:** 2026-06-22

**Authors:** Yi Wang, Rui Wang, Qi Zhu, Shuilin Liao, Yin Zhou, Shuting Xiao, Huaizhi Wang, Ziyue Zhang, Pijiang Sun, Qibin Song, Ming Dong

**Affiliations:** 1 Cancer Center, Renmin Hospital of Wuhan University, Wuhan, 430060 China; 2 Guangzhou National Laboratory, No. 9 XingDaoHuanBei Road, Guangzhou, 510005 China; 3 Cancer Center, Faculty of Health Sciences, University of Macau, Taipa, Macau SAR China; 4 State Key Laboratory of Respiratory Disease, The First Affiliated Hospital of Guangzhou Medical University, Guangzhou Medical University, Guangzhou, 510120 China; 5 Faculty of Innovation Engineering, Macau University of Science and Technology, Macau, 999078 China; 6 Institute of Hepatopancreatobiliary Surgery, Chongqing General Hospital, Chongqing University, Chongqing, 401147 China; 7 Chongqing Key Laboratory of Intelligent Medicine Engineering for Hepatopancreatobiliary Diseases, Chongqing, 401147 China; 8 Faculty of Health Sciences, University of Macau, Taipa, Macau SAR China; 9 University of Chinese Academy of Sciences (UCAS) Chongqing School, Chongqing Medical University, Chongqing, 401147 China; 10 Chongqing Institute of Advanced Pathology and Yu-Yue Scientific Research Center for Pathology, Jinfeng Laboratory, Chongqing, 401329 China

**Keywords:** Pancreatic cancer liver metastasis, RUNX1, Extracellular vesicle, ITGB1, Pre-metastatic niche, Hepatic stellate cell

## Abstract

**Background:**

Liver metastasis (LM) is the predominant distant metastatic target of pancreatic ductal adenocarcinoma (PDAC) and a major contributor to poor patient outcomes, with pre-metastatic niche (PMN) formation as its critical prerequisite. However, its mechanisms remain incompletely elucidated.

**Methods:**

RNA sequencing was performed on primary tumor tissues and paired adjacent non-tumor tissues from PDAC patients, combined with the analysis of a GEO dataset related to PDAC liver metastasis (PDAC-LM), followed by validation using in vitro assays and mouse orthotopic liver metastasis models. Transcription factor database screening coupled with dual-luciferase reporter assays identified downstream molecules. Mass spectrometry was conducted on plasma extracellular vesicles (EVs) from PDAC patients and healthy controls, with validation by ELISA. The hepatic effects of EVs were assessed using mouse models, and in vitro EV uptake assays were performed to dissect the underlying molecular mechanisms.

**Results:**

Transcriptomic sequencing of PDAC patients showed that elevated primary-tumor runt-related transcription factor 1 (RUNX1) expression correlates with PDAC-LM. In orthotopic murine models, RUNX1 knockdown significantly reduced liver metastasis rate from 61.54% to 8.33%. Mechanistically, RUNX1 transcriptionally upregulated integrin beta 1 (ITGB1), which was enriched in PDAC cell-secreted EVs and internalized by hepatic stellate cells (HSCs), activating the NF-κB pathway to induce HSC activation and secretion of ECM proteins, inflammatory factors and chemokines. This EV-induced HSC activation thereby promoted fibrotic hepatic PMN formation and facilitated LM, as validated in murine models. Clinically, ITGB1 in plasma EVs correlated with PDAC-LM and poor prognosis, with favorable diagnostic efficacy in distinguishing PDAC-LM patients.

**Conclusions:**

Here, we first demonstrate that ITGB1 upregulated by RUNX1 in primary PDAC cells is enriched in tumor-derived EVs and contributes to fibrotic PMN formation, thereby accelerating PDAC-LM. These pivotal findings not only unravel the intricate molecular circuitry governing PDAC-LM but also pinpoint promising biomarkers to facilitate the diagnosis and therapy of this lethal metastatic cascade.

**Supplementary Information:**

The online version contains supplementary material available at https://doi.org/10.1186/s12967-026-08521-3.

## Background

Pancreatic ductal adenocarcinoma (PDAC) remains one of the most lethal solid malignancies worldwide, with global incidence and mortality continuing to rise alongside the increasing prevalence of attributable metabolic and lifestyle risk factors [1, 2]. It is highly invasive and has a median overall survival time of 4 months across all stages of disease. Only 16% of patients are diagnosed with localized disease and eligible for curative surgical resection combined with chemotherapy, achieving a 5-year survival rate of 44% [3, 4]. However, 57% of patients present with distant metastases at diagnosis, most frequently in the liver (75–80% of metastatic lesions), leading to a drastically reduced 5-year survival probability of 3% [3, 4]. Yet effective approaches specifically targeting PDAC liver metastasis (PDAC-LM) remain limited, lagging behind the progressive therapeutic advances achieved for primary hepatic malignancies arising within the same hepatic microenvironment, owing to its distinct biological features and the lack of well-defined mechanistic targets governing hepatic metastatic colonization [5–8]. There is therefore an urgent need to further clarify molecular and pathological regulatory mechanisms underlying PDAC-LM and formulate more potent therapeutic strategies targeting this lethal disease.

As a sophisticated multi-stage biological cascade, tumor metastasis involves malignant cells disseminating from primary tumor lesions to distant organs and developing secondary metastatic foci [9]. Underpinned by the seed-and-soil hypothesis [10, 11], crosstalk between tumor cells and the target-organ microenvironment is pivotal for successful metastatic colonization [12]. Central to this crosstalk is the pre-metastatic niche (PMN)—a permissive microenvironment that supports the homing and colonization of disseminated tumor cells at distant secondary loci, characterized by key features including immunosuppression, angiogenesis/vascular hyperpermeability, lymphangiogenesis, extracellular matrix (ECM) reprogramming, organotropism, and inflammation [13, 14]. Accumulating evidence indicates that primary tumors can trigger metastatic processes by inducing PMN formation in distant organs, which is mediated by the secretion of soluble factors including secreted proteins, cytokines, and extracellular vesicles (EVs) [15].

Among these mediators, tumor-derived EVs stand out as critical carriers for long-distance crosstalk between primary tumors and target organs, playing a pivotal regulatory role in PMN establishment [16]. These EVs encapsulate various bioactive molecules, including microRNAs, lipids, and proteins, which are delivered to the target organs via systemic circulation to reshape the local microenvironment by modulating the functions of resident cells [17–21]. While emerging studies confirm that tumor-derived EVs from various primary tumors contribute to intrahepatic PMN formation, how the cargo composition of PDAC-derived EVs is regulated, and how such EV cargo engages downstream stromal cell populations to license hepatic colonization, remain largely uncharacterized in PDAC-LM.

Runt-related transcription factor 1 (RUNX1), a key transcription factor implicated in cancer progression, exhibits distinct roles in tumor metastasis in a cancer-type-dependent manner. It promotes metastasis in cancers such as hepatocellular carcinoma and colorectal cancer, while exerting tumor-suppressive effects in gastric and breast cancers, with its function relying on specific downstream signaling pathways [22–24]. In PDAC, RUNX1 has been confirmed to exert a pivotal role in its malignant progression [25–27]. However, whether RUNX1 contributes to PDAC liver metastasis, and the molecular mechanisms underlying such a role, remain unexplored.

Motivated by these unresolved questions, the present study demonstrates that RUNX1-driven elevation of integrin beta 1 (ITGB1) in tumor-derived EVs contributes to PDAC liver metastasis by promoting intrahepatic fibrotic PMN formation. By directly linking a primary-tumor transcription factor to EV cargo composition and downstream stromal activation, our findings delineate a previously unrecognized RUNX1–EV–ITGB1 axis, and provide valuable insights for developing novel diagnostic, prognostic and therapeutic strategies against this devastating disease.

## Materials and methods

### Clinical specimens

In this study, 10 pairs of non-metastatic primary PDAC tissues and para-cancerous tissues, as well as 6 primary PDAC tissues from patients with liver metastasis were obtained from Chongqing People’s Hospital. The plasma samples of patients with PDAC or healthy controls were also collected from Chongqing People’s Hospital. This study was approved by the Ethics Committee of Chongqing People’s Hospital, in compliance with the Declaration of Helsinki. All samples were collected after obtaining written informed consent from the subjects.

### Bioinformatics analyses of clinical data

Gene expression microarray datasets of human-originated pancreatic cancer (GSE71729) were retrieved from the Gene Expression Omnibus (GEO) database. Online analysis tools BEST (https://rookieutopia.hiplot.com.cn/app_direct/BEST/) and GEPIA (http://gepia.cancer-pku.cn/) were used for clinical association, gene expression correlation, and survival analyses of PDAC. TF-Target Finder, a novel integrative tool [28] designed to decode transcription factor–target interactions was employed to predict target genes of RUNX1.

### Cell lines and cell culture

Cell lines were obtained from the Cell Resource Center, Institute of Biochemistry and Cell Biology at the Chinese Academy of Science (Shanghai, China). MIA PaCa-2, PANC-1, Pan02, hTERT-HPNE, LX-2 and 293T cells were cultured in DMEM. SW1990, ASPC-1, BxPC-3 and HPAF-2 were cultured in RPMI 1640 cell culture medium. CFPAC-1 was cultured in IMDM. All culture media were supplemented with 10% fetal bovine serum (FBS) and 1% penicillin-streptomycin. All cell lines were authenticated and confirmed to be mycoplasma-free, and were maintained at 37 °C in a humidified atmosphere of 5% CO_2_.

### RNA extraction and qRT-PCR

Total RNA was extracted from cells and tissues, reverse-transcribed into cDNA, and amplified by quantitative PCR using reagents from Vazyme (China). Primer sequences are presented in Supplementary Table 1. Relative mRNA expression was quantified by the 2⁻ΔΔCt method and normalized to 18 S rRNA or GAPDH.

### Western blot (WB)

Cultured cells were subjected to lysis with RIPA buffer (Beyotime, China). 6–10% gradient SDS-polyacrylamide gels were used for electrophoresis separation of protein samples. Separated proteins were transferred to polyvinylidene fluoride membranes (Millipore, USA), blocked, incubated with primary and secondary antibodies, and target bands detected by the ECL kit (ServiceBio, China). Full details of antibodies employed are presented in Supplementary Table 2.

### Lentivirus production

Short hairpin RNAs (shRNAs) targeting RUNX1, ITGB1, and negative control were cloned into the lentiviral vector pLKO.1 (Addgene, USA). Recombinant plasmids and their inserted sequences were verified by DNA sequencing. Subsequently, each recombinant vector was separately co-transfected into 293T cells with a lentiviral packaging system using PEI Transfection Reagent (MedChemExpress, USA). CFPAC-1 and Pan02 cells underwent lentiviral infection with polybrene added (Beyotime, China), followed by selection with puromycin. shRNA sequences are listed in Supplementary Table 3.

### CCK-8 assay

96-well plates were seeded with 4000 cells per well at a uniform density. Each well received 10 µL of CCK-8 reagent (Goonie, China), followed by incubation at 37 ℃ for 1 h. A microplate reader was used to quantify the absorbance at 450 nm thereafter.

### Wound-healing assay

Target cells were plated into 6-well culture plates and maintained until full confluence was achieved. Linear scratches were made on the confluent cell monolayer. Loosely adherent cells were eliminated via two rounds of gentle rinsing with serum-free medium, followed by cell incubation in 2% FBS-supplemented medium at 37 °C for 48 h. Cell images were acquired at 0 h (baseline) and 48 h. Wound areas corresponding to these two time points were measured and analyzed with ImageJ software.

### Transwell assay

5 × 10⁴ serum-free cells were plated in the upper of Matrigel-precoated Transwell chambers (Corning, USA), with 600 µL 10% FBS medium in the lower. After incubation at 37 °C for 36–48 h, cells on the lower surface were fixed in methanol and stained with 5 g/L crystal violet for 10 min. Three random fields were chosen for photographing and statistical analysis.

### Mice and tumor models

Female wild-type C57BL/6J mice (6–8 weeks old, immunocompetent) were used in this study. All experimental protocols were approved by the Institutional Animal Care and Use Committee (IACUC) of Guangzhou National Laboratory and conducted in accordance with the NIH Guide for the Care and Use of Laboratory Animals. To establish the pancreatic orthotopic tumor-induced liver metastasis model, a 1 cm left-flank incision was made, and the pancreas with spleen was exteriorized and flattened. Then, 2 × 10^6^ Pan02 cells (resuspended in 25 µL Matrigel) were injected into the pancreatic parenchyma. For the EV-educated pancreatic orthotopic injection-induced liver metastasis model, naive wild-type mice received 20 µg Pan02-derived EVs in 200 µL PBS via tail vein injection twice weekly for 3 weeks. Following EVs education, 2 × 10^6^ Pan02 cells were orthotopically injected into the pancreas as described above, and liver metastasis incidence was subsequently analyzed. To evaluate the effects of PDAC-derived EVs on the hepatic pre-metastatic niche, mice received PBS, shNC-EVs, or shITGB1-EVs via tail vein injection (20 µg in 200 µL PBS per mouse, twice weekly for 3 weeks), and livers were harvested for subsequent analyses.

### Immunofluorescence (IF)

For IF staining, deparaffinization was first performed on formalin-fixed paraffin-embedded (FFPE) sections, followed by antigen retrieval in retrieval solution (Biosharp, China) using a microwave for 20 min, while these steps were omitted for cryosections. Cultured cells were fixed in 4% paraformaldehyde and underwent permeabilization with 0.25% Triton X-100. 5% BSA-blocked samples were incubated with primary (4 °C/overnight) and secondary (RT/2 h) antibodies, DAPI-stained, and visualized via ZEISS confocal system. Antibody information is listed in Supplementary Table 2.

### Luciferase reporter assay

The ITGB1 promoter region spanning from − 2000 to + 100 bp was subcloned into the pGL3-basic luciferase reporter vector. HEK293T cells were co-transfected with 0.3 µg pGL3-ITGB1-promoter, 0.3 µg RUNX1 expression plasmid (pcDNA3.1-RUNX1), and 0.06 µg pRL-SV40P (internal control) using Lipofectamine 3000 (Invitrogen, USA). The empty vector pcDNA3.1-NC served as the negative control. Additionally, CFPAC-1 cells with stable RUNX1 knockdown or scramble control were co-transfected with 0.5 µg pGL3-ITGB1-promoter and 0.05 µg pRL-SV40P. For both cell types, cells were subjected to lysis with passive lysis buffer at 48 h post-transfection. Luciferase activity was assayed using a Dual-Luciferase Reporter Assay kit (Promega, USA) on a multi-mode microplate reader (Bio Tek, SYNERGY NEO2). Renilla luciferase activity served as the internal reference to normalize firefly luciferase activity.

### EV isolation and characterization

EVs were isolated and purified via sequential centrifugation. Briefly, two sample types were processed, including plasma from PDAC patients and healthy individuals, as well as pancreatic cancer cell culture medium from cells cultured in 10% EV-free FBS for 48 h. Both samples were processed by three-step centrifugation with conditions set as 300 × g for 10 min, 2,000 × g for 10 min, and 10,000 × g for 30 min, and the resulting supernatant was passed through 0.22-µm polyethersulfone filters (Millipore, USA). Thereafter, EV-containing pellets were harvested by ultracentrifugation at 100,000 × g for 70 min. Following a PBS rinse, the EVs were collected via a second round of ultracentrifugation under the same conditions.

EV characterization was performed via transmission electron microscopy (TEM, Thermo Fisher Scientific, Talos L120C G2). EV concentration and size distribution were measured using nanosight particle tracking analysis (NTA, Malvern Panalytical, NS300). Total protein concentration was measured by BCA assay. Particle-to-protein ratios were calculated as the number of particles per microgram of protein.

### ELISA

ELISA (Enzyme-linked immunosorbent assay) kits (JONLNBIO, China) were used to quantify the protein levels of mouse ITGB1 and human ITGB1 in EVs isolated from serum or plasma samples. Signal detection was performed at 450 nm using a microplate reader.

### Proteomic analysis

Proteomic profiling was carried out at Guangzhou National Laboratory. Plasma EVs isolated from PDAC patients and healthy controls underwent ultrasonic lysis with 1% protease inhibitor. After protein digestion, peptides were desalted and lyophilized, and LC-MS/MS datasets analyzed via MaxQuant (v1.5.3.8) for protein identification.

### EV labeling and tracing

For in vivo tracking, Pan02 cell-derived EVs were labeled with DiD dye (MedChemExpress, USA). Briefly, DiD was incubated with isolated EVs at a final working concentration of 5 µM at 37 °C for 30 min, followed by PBS rinsing and ultracentrifugation at 100,000 × g for 70 min to eliminate unbound excess dye. DiD-labeled EVs (~ 20 µg in 200 µL PBS per mouse) were administered to C57BL/6J mice by tail vein injection. Tissue collection and analysis of EV distribution were performed 24 h later using the IVIS imaging system (Biolight Biotechnology, AniView100 Pro) and immunofluorescence.

For in vitro assessment of EVs uptake by LX-2 cells, DiD-labeled EVs were added to LX-2 culture medium and co-incubated with cells at 37 °C for 6 h, followed by staining with DAPI and a desmin antibody.

### EV treatment of LX-2 cells

LX-2 cells were starved in serum-free medium for 12 h and then treated with PBS, shNC-EVs, or shITGB1-EVs at 100 µg/mL (based on EV protein quantified by BCA assay) for 48 h, followed by qRT-PCR, Western blot, or immunofluorescence analysis. For the NF-κB inhibitor rescue assay, LX-2 cells were pretreated with 7.5 µM BAY 11-7082 (MedChemExpress, USA) for 2 h prior to shNC-EV stimulation.

### Statistical analysis

Statistical analyses were performed using R (v 4.3.1), SPSS (v 22.0), and GraphPad Prism (v 9.5.1). Experiments were conducted with at least three independent biological replicates, and quantitative data are presented as mean ± standard deviation (SD). Data distribution and variance homogeneity were assessed prior to between-group comparisons. Two-group comparisons were performed using the unpaired two-tailed Student’s t test for normally distributed data with equal variances, or the Mann–Whitney U test otherwise. Multi-group comparisons were performed using one-way ANOVA followed by Tukey’s or Dunnett’s post hoc test when the above assumptions were satisfied, or the Kruskal–Wallis test with Dunn’s post hoc test otherwise. Categorical variables were analyzed using Fisher’s exact test. *P* values from RNA sequencing and mass spectrometry datasets were adjusted using the Benjamini–Hochberg procedure. Survival outcomes were assessed by the Kaplan–Meier method with log-rank testing. A two-sided *P* < 0.05 was considered statistically significant (*P < 0.05, **P < 0.01, ***P < 0.001; n.s., not significant).

## Results

### Elevated RUNX1 expression is related to liver metastasis and unfavorable prognosis in PDAC patients

**Fig. 1:**
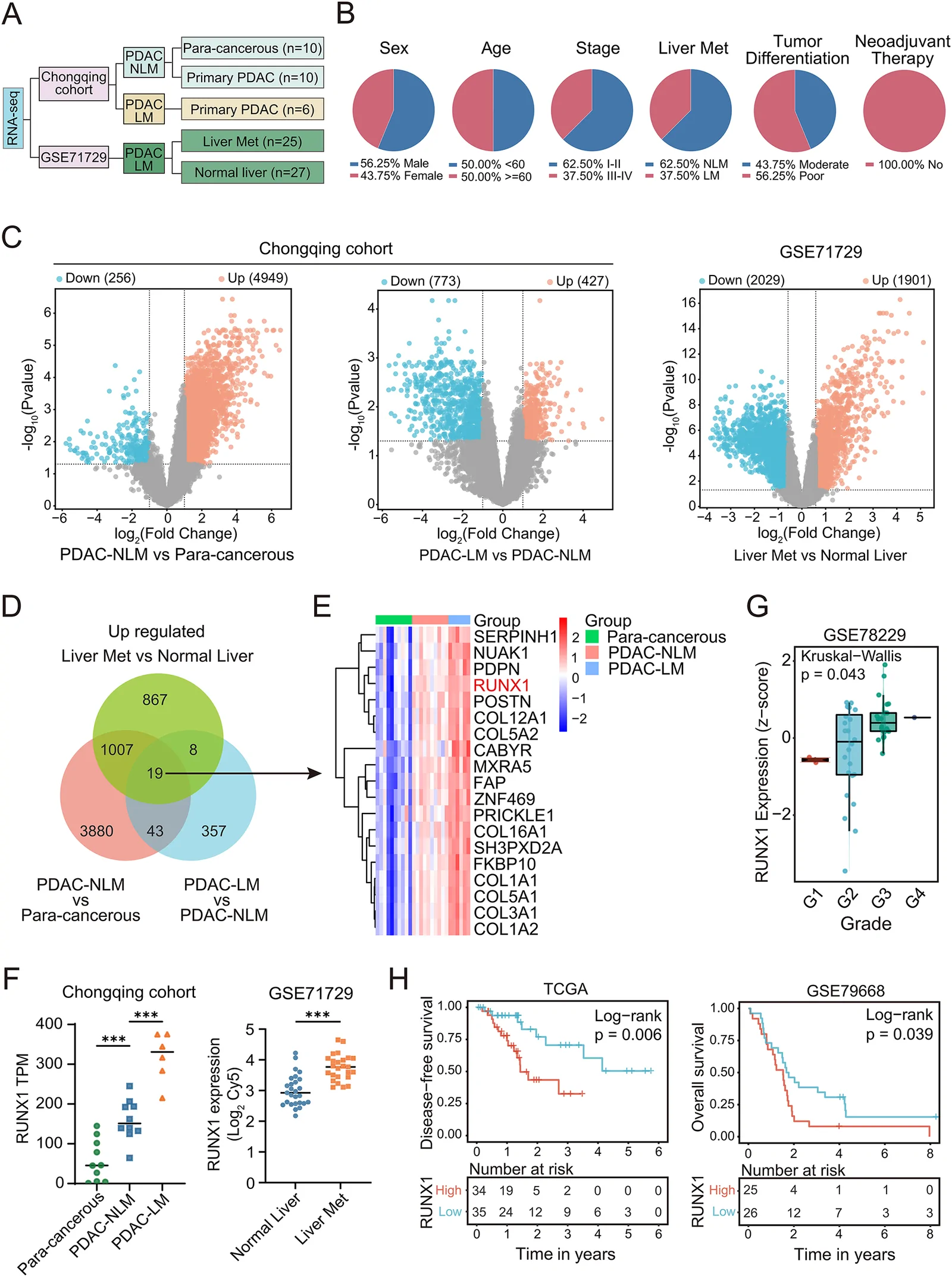
RUNX1 is upregulated in PDAC with liver metastases and associates with poor prognosis. (**A**) Schematic diagram of the data sources. (**B**) Main clinical features of PDAC patients from Chongqing cohort. (**C**) Volcano plots for transcriptomic differential gene analysis of primary pancreatic tumors and paired para-cancerous tissues of 10 PDAC-NLM patients, primary tumors of 6 PDAC-LM patients from Chongqing People’s Hospital, 25 PDAC liver metastases and 27 normal liver tissues in GSE71729. (**D**) Venn diagram of differentially upregulated genes derived from Chongqing cohort and GSE71729. (**E**) The heat map of 19 common overexpressed genes. (**F**) mRNA expression of RUNX1 in para-cancerous tissues of PDAC-NLM (*n* = 10), primary tumors of PDAC-NLM (*n* = 10), and primary tumors of LM patients (*n* = 6) from the Chongqing cohort, and in liver metastases (*n* = 25) and normal liver tissues (*n* = 27) from GSE71729. (**G**) The correlation between RUNX1 expression and the tumor grade of pancreatic cancer in GSE78229. (**H**) Kaplan–Meier plots show the disease-free survival of PDAC patients from the TCGA dataset and overall survival from GSE79668 dataset based on RUNX1 mRNA expression. Data are shown as mean ± SD. Unpaired Student’s t-test, one-way ANOVA followed by Tukey’s post-hoc test, Kruskal–Wallis test, and log-rank test were used. ***P < 0.001

To investigate transcriptome alterations influencing PDAC-LM and its microenvironment, RNA sequencing was performed on primary tumors and paired para-cancerous tissues from 10 PDAC patients without liver metastasis (PDAC-NLM), as well as on primary tumors from 6 PDAC patients with liver metastasis (PDAC-LM), all sourced from Chongqing People’s Hospital. Additionally, a GEO dataset (GSE71729) containing bulk-tissue transcriptome data of 25 PDAC liver metastatic tissues and 27 normal liver tissues was analyzed (Fig. 1A). The main clinical characteristics of patients from Chongqing cohort were summarized in Fig. 1B, including age, sex, TNM stage, presence of liver metastasis, tumor differentiation grade, and neoadjuvant treatment status. Notably, all tissue specimens were obtained from treatment-naive patients who had not received neoadjuvant therapy prior to tissue procurement. A detailed comparison of baseline demographics and clinicopathological variables between the PDAC-NLM and PDAC-LM groups is presented in Supplementary Table 4. No significant differences were identified except for tumor size, confirming the overall comparability of the two groups. Differential analysis across these datasets identified 19 significantly upregulated genes, mostly associated with fibrosis (Fig. 1C–E). Interestingly, among the upregulated genes identified, RUNX1 has previously been implicated in promoting pancreatic cancer progression [26, 27] and is also recognized for its significant role in colorectal cancer liver metastasis [23, 24]. Nevertheless, its specific function in PDAC-LM is not yet clarified. Consequently, we focused our research on investigating RUNX1. RUNX1 expression was significantly higher in primary tumors from PDAC-NLM patients than in matched para-cancerous tissues, and was further elevated in primary tumors from PDAC-LM patients (Fig. 1F). GSE71729 dataset analysis revealed markedly higher RUNX1 expression in liver metastatic tissues versus normal hepatic tissues (Fig. 1F). In addition, RUNX1 expression exhibited a positive correlation with the tumor grade of pancreatic cancer in GSE78229 (Fig. 1G). Moreover, analysis of the TCGA and GSE79668 datasets revealed that high RUNX1 expression was associated with unfavorable prognosis regarding overall survival (OS) and disease-free survival (DFS) among PDAC patients (Fig. 1H). Taken together, these observations indicate that elevated RUNX1 expression correlates with PDAC-LM and is associated with unfavorable prognosis.

### RUNX1 facilitates proliferation, migration and invasion of pancreatic cancer cells in vitro

**Fig. 2:**
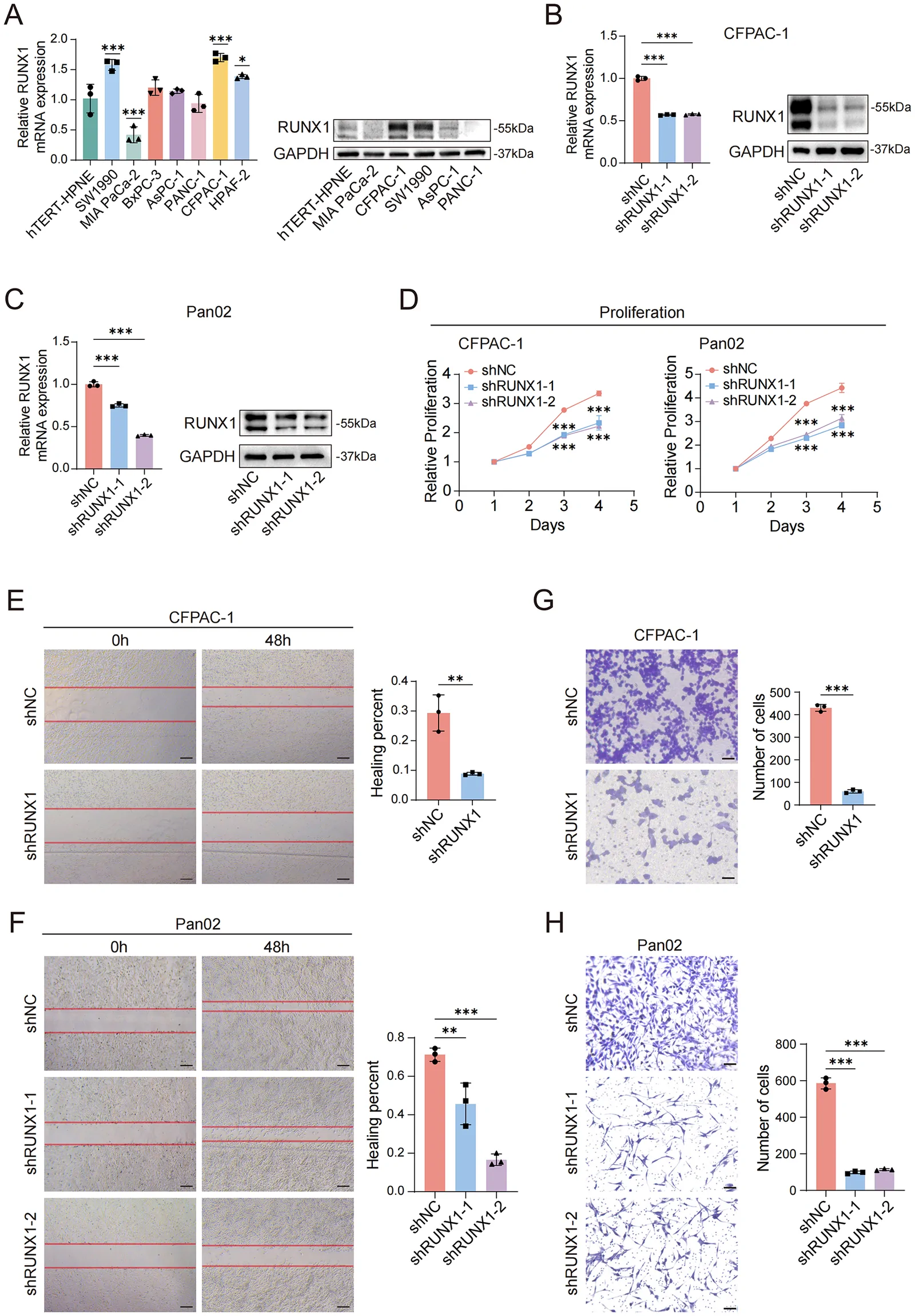
RUNX1 promotes PDAC cell proliferation, migration, and invasion in vitro. (**A**) qPCR and WB analyses of RUNX1 expression in different pancreatic cell lines. (**B**, **C**) The validation of RUNX1 knockdown in CFPAC-1 and Pan02 by qRT-PCR and WB. (**D**) CCK-8 assay illustrating the proliferative capacity of RUNX1-knockdown pancreatic cancer cells. (**E**, **F**) Wound-healing assay assessing cell migration. Scale bar, 500 μm. (**G**, **H**) Transwell assays served to assess the invasive capacity of cells. Scale bar, 200 μm. Data are presented as mean ± SD. Unpaired Student’s t-test and one-way ANOVA followed by Dunnett’s test were used. ns, not significant (*P* ≥ 0.05); *P < 0.05, **P < 0.01, ***P < 0.001

To explore RUNX1 expression, we performed qRT-PCR and WB on several human pancreatic cancer cell lines and one normal pancreatic ductal epithelial cell line (HPNE). The highest RUNX1 expression was observed in CFPAC-1 cells (Fig. 2A). To assess the function of RUNX1 during PDAC progression, CFPAC-1 and murine pancreatic cancer cell line Pan02 were established with stable RUNX1 knockdown using shRNAs (Fig. 2B, C). Relative to the control group, RUNX1 knockdown led to suppressed proliferation of CFPAC-1 and Pan02 cells, as determined by the CCK-8 assay (Fig. 2D). Using the wound-healing assay, we discovered that RUNX1 downregulation significantly decreased pancreatic cancer cell migration capacity (Fig. 2E, F). The transwell assay demonstrated that RUNX1 facilitated pancreatic cancer cell invasion (Fig. 2G, H). Collectively, these findings indicate that RUNX1 plays an important role in pancreatic cancer cell proliferation, migration, and invasion in vitro.

### RUNX1 promotes liver metastasis of PDAC in vivo

**Fig. 3:**
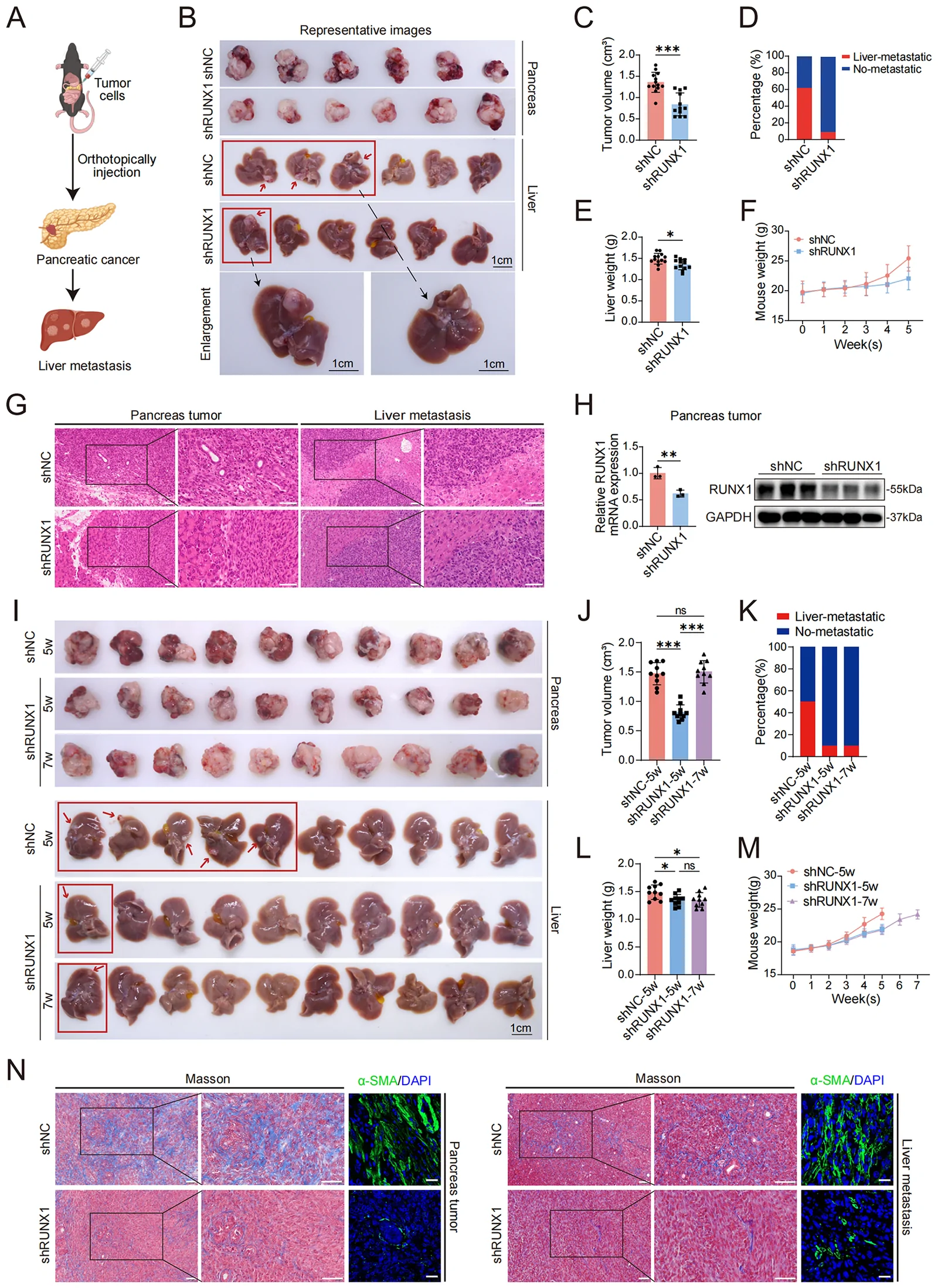
RUNX1 promotes liver metastasis of PDAC in vivo. (**A**) Schematic diagram of pancreatic orthotopic tumor-induced liver metastasis model. (**B**) Representative photograph of pancreatic orthotopic tumors and liver metastases. (**C**-**E**) Orthotopic tumor volume, liver metastasis rate, and liver weight were measured (shNC, *n* = 13; shRUNX1, *n* = 12). (**F**) Change of mouse weight over time. (**G**) Representative H&E staining images of pancreatic orthotopic tumors and liver metastases (Scale bar, 50 μm). (**H**) qRT-PCR and Western blot validation of reduced RUNX1 expression in orthotopic tumors of the RUNX1 knockdown group compared with controls. (**I**) Gross images of pancreatic orthotopic tumors and livers harvested from the shNC-5w, shRUNX1-5w, and shRUNX1-7w groups (*n* = 10 per group). (**J**-**L**) Comparison of orthotopic tumor volumes, liver metastasis rates, and liver weights of mice in the shNC-5w, shRUNX1-5w, and shRUNX1-7w groups. (**M**) Mouse weight curves over the observation period. (**N**) Representative Masson’s trichrome staining (Scale bar, 50 μm) and IF staining for α-smooth muscle actin (α-SMA) (Scale bar, 20 μm) in pancreatic orthotopic tumors and liver metastases. Data are presented as mean ± SD. Unpaired Student’s t-test and one-way ANOVA followed by Tukey’s post-hoc test was used for statistical analysis. *P < 0.05, **P < 0.01, ***P < 0.001, n.s., not significant

To investigate the role of RUNX1 in PDAC progression in vivo, a pancreatic orthotopic tumor-induced liver metastasis model was established in C57BL/6J mice by injecting Pan02 cells with stable RUNX1 knockdown and control cells into the pancreas. Six weeks after orthotopic pancreatic injection, mice were humanely euthanized (Fig. 3A). The volume of orthotopic tumors was markedly decreased in the RUNX1-knockdown group versus the control group (Fig. 3B, C). Consistently, RUNX1 knockdown significantly reduced the liver metastasis rate from 61.54% to 8.33% (Fig. 3B, D). In addition, the liver weight in the RUNX1 knockdown group was significantly diminished compared to the control group (Fig. 3E). From three weeks after injection onward, mice in the control group exhibited markedly higher body weight than those in the RUNX1-knockdown group, likely reflecting accelerated tumor growth and the more abundant ascites observed upon dissection (Fig. 3F). H&E staining further confirmed the presence of pancreatic orthotopic tumors and corresponding liver metastases across the experimental cohorts (Fig. 3G). qRT-PCR and WB confirmed lower RUNX1 expression in pancreatic orthotopic tumors of the RUNX1 knockdown group versus the control group (Fig. 3H).

Although the above findings demonstrated that RUNX1 knockdown concurrently attenuated primary tumor growth and hepatic metastasis, it remained unclear whether the reduction in liver metastasis rate was simply a passive consequence of diminished primary tumor burden, or whether RUNX1 exerted an additional, tumor size–independent effect on the metastatic process per se. To dissect these two possibilities, we designed a tumor volume–matched experiment in which Pan02-shNC and Pan02-shRUNX1 cells were orthotopically implanted into C57BL/6J mice, and animals were sacrificed at two distinct time points: shNC mice at 5 weeks (shNC-5w, *n* = 10) and shRUNX1 mice at both 5 weeks (shRUNX1-5w, *n* = 10) and 7 weeks (shRUNX1-7w, *n* = 10) post-implantation. The extended observation window for the shRUNX1 group was intended to allow the slower-growing knockdown tumors sufficient time to reach a volume comparable to that of the shNC-5w group, thereby decoupling primary tumor size from metastatic propensity (Fig. 3I). As anticipated, at 5 weeks post-implantation, orthotopic tumor volume in the shNC group substantially exceeded that of the shRUNX1 group, whereas by 7 weeks the shRUNX1 tumors had grown to a size comparable to the 5-week shNC tumors, confirming successful volume matching between the shNC-5w and shRUNX1-7w cohorts (Fig. 3J). Strikingly, although the shRUNX1-7w group bore primary tumors of equivalent volume to the shNC-5w group, its liver metastasis rate remained low (10%), nearly identical to that of the shRUNX1-5w group (10%) and markedly lower than that of the shNC-5w group (50%) (Fig. 3K). Consistently, liver weights were significantly higher in shNC-5w mice than in either shRUNX1 cohort, with no difference between the two knockdown groups (Fig. 3L). Body weight trends paralleled these findings (Fig. 3M). The persistently low metastatic rate in the shRUNX1-7w group, despite tumor volumes matching those of the shNC-5w group, suggests that RUNX1 knockdown suppresses liver metastasis not only by reducing primary tumor burden but also through an additional, tumor size–independent effect on the metastatic cascade itself. Considering that most of the common differentially expressed genes (DEGs) identified in our transcriptome analysis were associated with fibrosis, we further examined the fibrotic status of pancreatic orthotopic tumors and liver metastases by Masson’s trichrome and immunofluorescence (IF) staining. The control group displayed markedly more pronounced fibrotic deposition than the RUNX1 knockdown group at both the orthotopic and metastatic sites (Fig. 3N). Collectively, these in vivo findings demonstrate that RUNX1 promotes PDAC primary tumor growth, fibrotic remodeling, and hepatic metastasis through mechanisms beyond primary tumor burden alone.

### RUNX1 regulates ITGB1 as a target gene to promote PDAC malignancy

**Fig. 4:**
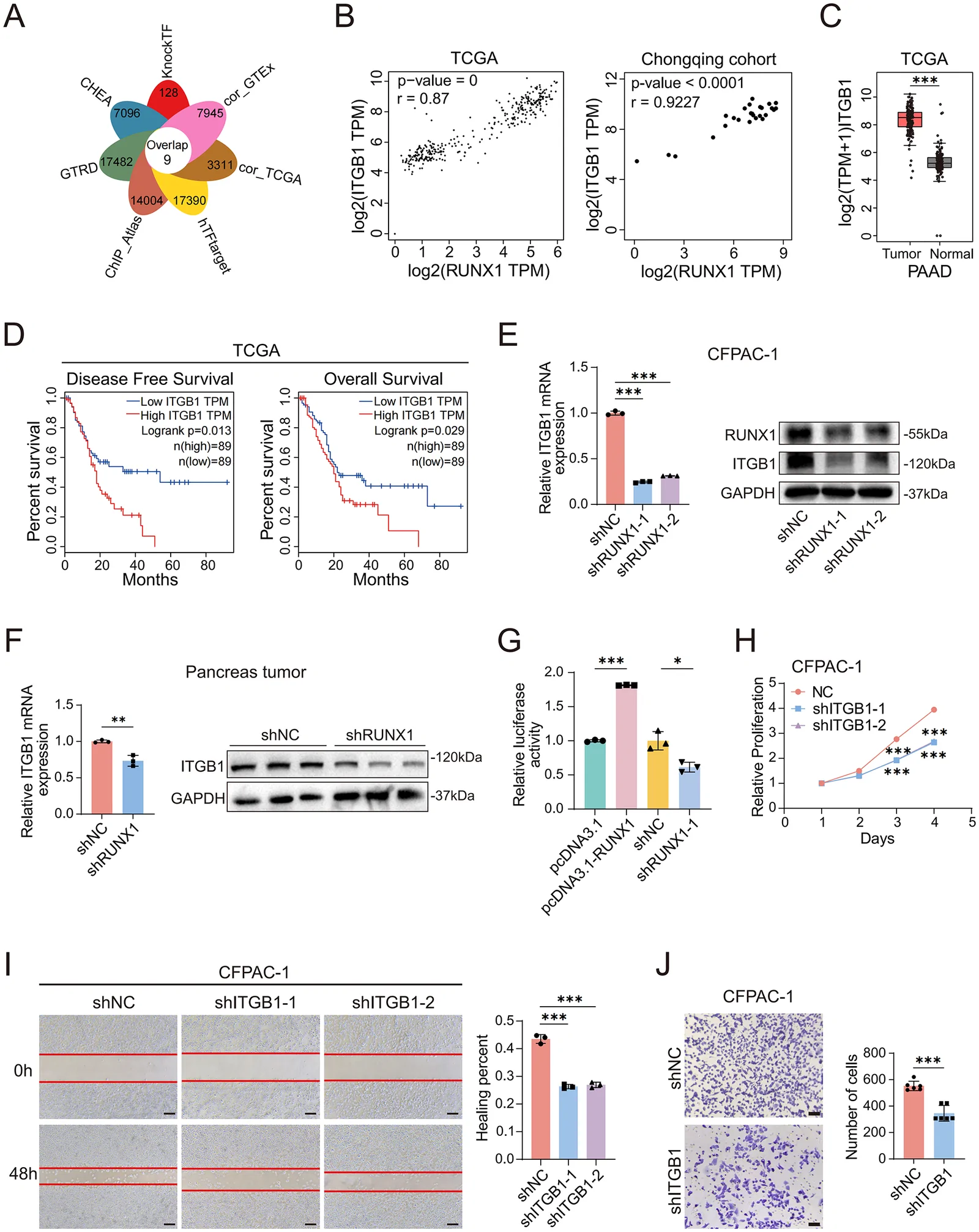
RUNX1 regulates ITGB1 as a target gene to promote PDAC malignancy. (**A**) A petal diagram illustrating RUNX1 target genes from five major transcription factor databases and two correlation analyses. (**B**) The correlation of ITGB1 and RUNX1 mRNA expression in PDAC from TCGA dataset and Chongqing cohort. (**C**) ITGB1 expression in PDAC and normal pancreatic tissues from TCGA dataset. (**D**) Kaplan–Meier plots showing the DFS and OS of PDAC patients from the TCGA dataset stratified by ITGB1 mRNA expression. (**E**) ITGB1 expression in RUNX1-knockdown CFPAC-1 cells. (**F**) ITGB1 expression in pancreatic orthotopic tumors of the mouse model. (**G**) ITGB1 promoter activity was evaluated by dual-luciferase reporter assay in cells with RUNX1 overexpression or knockdown. (**H**) CCK-8 assay demonstrating the impact of ITGB1 knockdown on proliferation in CFPAC-1 cell. (**I**) Migration ability was assessed by a wound-healing assay. Scale bar, 500 μm. (**J**) Transwell assay was used to assess the cell invasive ability. Scale bar, 200 μm. Data are presented as mean ± SD. Statistical analyses were performed using Pearson correlation coefficient, unpaired Student’s t-test, one-way ANOVA followed by Tukey’s post-hoc test, and log-rank test. *P < 0.05, **P < 0.01, ***P < 0.001

To further elucidate the mechanisms underlying RUNX1-mediated PDAC liver metastasis, we used TF-Target Finder [28], a transcription factor target prediction web tool, to identify potential target genes of RUNX1. By integrating five major transcription factor databases (hTFtarget, KnockTF, CHEA, GTRD, and CHIP-Atlas), along with correlation analyses of RUNX1 with other genes in PDAC (TCGA) and pancreatic tissues (GTEx), we identified nine potential target genes (Fig. 4A, Supplementary Fig. 1A). Notably, among these nine candidates, ITGB1 expression exhibited strong positive correlation with RUNX1 not only in TCGA dataset (*r* = 0.87, *P* < 0.0001) but also in our Chongqing cohort (*r* = 0.9227, *P* < 0.0001) (Fig. 4B). Furthermore, RUNX1 and ITGB1 expression was significantly positively correlated across most cancer types and normal tissues (Supplementary Fig. 1B). Moreover, ITGB1 expression was significantly higher in PDAC tissues compared with normal pancreatic tissues (Fig. 4C, Supplementary Fig. 1C). Importantly, TCGA-based survival analysis revealed that PDAC patients with lower ITGB1 mRNA expression exhibited prolonged OS and DFS (Fig. 4D). Meanwhile, RUNX1 knockdown significantly reduced ITGB1 expression in both CFPAC-1 cells and pancreatic orthotopic tumors of the mouse model (Fig. 4E, F). These findings indicate that ITGB1 is strongly correlated with RUNX1 and may further play an important role in the progression of PDAC. To delineate how RUNX1 modulates ITGB1 expression, we generated a dual-luciferase reporter vector harboring the ITGB1 promoter. ITGB1 promoter activity was found to be enhanced by RUNX1 via dual-luciferase reporter assays, whereas this activation was attenuated following RUNX1 knockdown (Fig. 4G), indicating that RUNX1 regulates ITGB1 transcription via promoter activation, thereby modulating ITGB1 mRNA and protein levels.

To validate ITGB1 as a functional downstream target of RUNX1, we established stable ITGB1 knockdown CFPAC-1 and Pan02 cell lines (Supplementary Fig. 2A). Compared with controls, ITGB1 knockdown inhibited the proliferation, migration, and invasion of CFPAC-1 and Pan02 cells (Fig. 4H-J, Supplementary Fig. 2B-D). Collectively, these findings support that ITGB1 acts as a RUNX1 downstream effector to promote PDAC progression.

### ITGB1 is upregulated in liver metastatic PDAC EVs and correlated with unfavorable prognosis

**Fig. 5:**
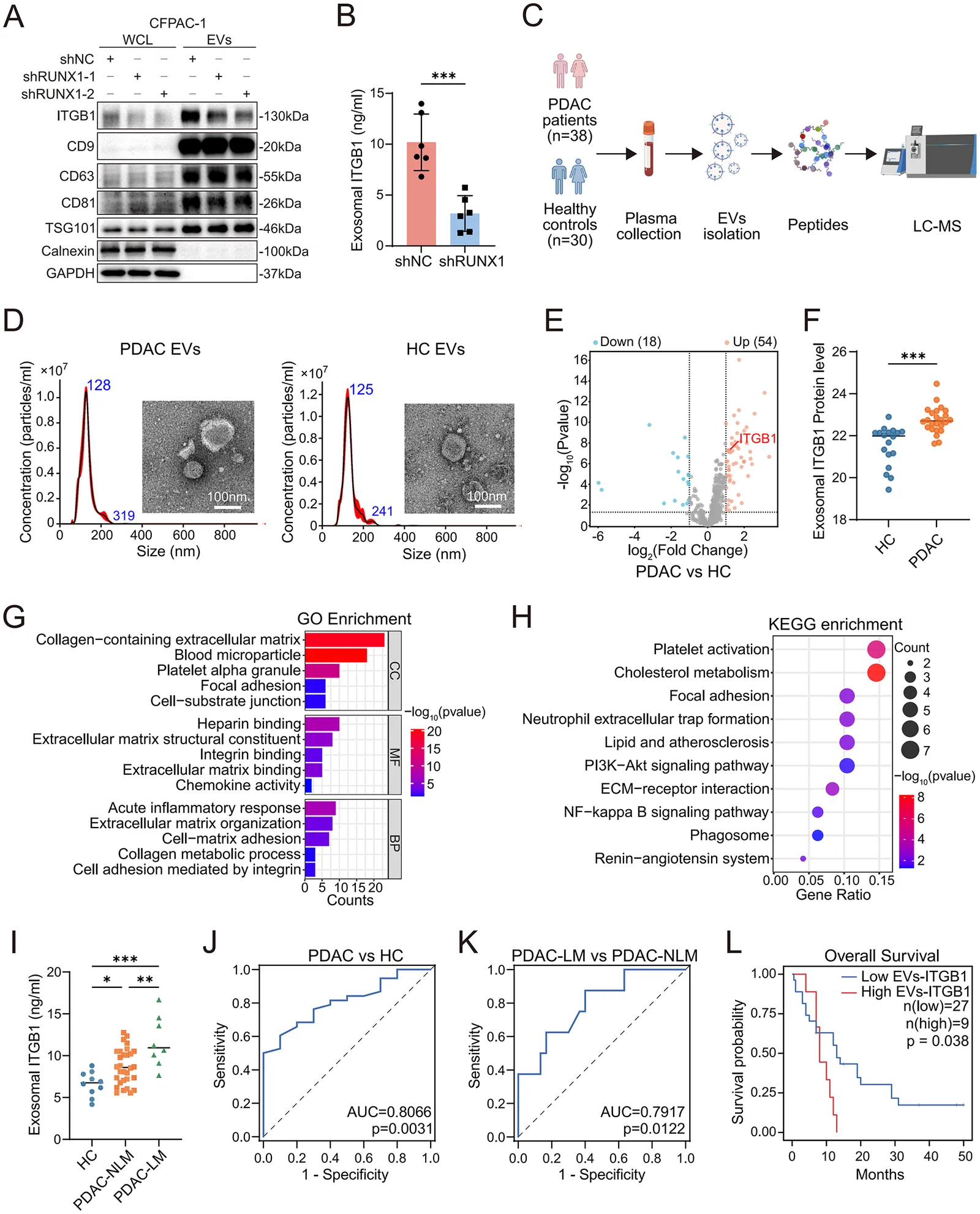
ITGB1 is upregulated in liver metastatic PDAC EVs and correlated with unfavorable prognosis. (**A**) Western blot analysis of canonical EV markers and ITGB1 in EVs and paired whole-cell lysates (WCL) from shNC and shRUNX1 CFPAC-1 cells. (**B**) ITGB1 levels in plasma EVs from mice bearing pancreatic orthotopic tumors, as detected by ELISA (*n* = 6). (**C**) Schematic workflow of EVs mass spectrometry. (**D**) Characterization of plasma EVs from PDAC patients and healthy controls using TEM and NTA. (**E**) Volcano plot of differentially expressed proteins in plasma EVs from PDAC patients and healthy controls. (**F**) ITGB1 levels in plasma EVs from healthy controls (*n* = 30) and PDAC patients (*n* = 38). (**G**-**H**) GO and KEGG pathway analysis of differentially expressed proteins. (**I**) ITGB1 levels in plasma EVs of healthy controls (*n* = 10), PDAC-NLM patients (*n* = 30), and PDAC-LM patients (*n* = 8) detected by ELISA. (**J**) ROC curve of plasma EV-derived ITGB1 between PDAC patients and healthy controls. (**K**) ROC curve of plasma EV-derived ITGB1 between PDAC-LM and PDAC-NLM. (**L**) Kaplan–Meier plots showing the OS of PDAC patients from Chongqing People’s Hospital stratified by EVs ITGB1 level measured by mass spectrometry. Data are presented as mean ± SD. Statistical analyses were performed using unpaired Student’s t-test, one-way ANOVA followed by Tukey’s post-hoc test, and log-rank test. *P < 0.05, **P < 0.01, ***P < 0.001

Tumor-derived EVs are critical mediators of distant metastasis, and ITGB1 is a ubiquitously expressed, functionally important protein on EVs. Additionally, ITGB1 and other integrins have been shown to be transported via EVs to facilitate tumor metastasis [29, 30]. Thus, building on the above findings that RUNX1 directly regulates intracellular ITGB1 as its downstream target, we hypothesized that RUNX1 may regulate ITGB1 level in EVs to drive PDAC-LM. To test this hypothesis, EVs were first isolated from shRUNX1 and shNC CFPAC-1 cells and subjected to comprehensive characterization, including TEM, NTA, Western blot detection of canonical positive and negative EV markers, and EV particle-to-protein ratio quantification (Fig. 5A, Supplementary Fig. 3A, Supplementary Table 5). Both preparations exhibited typical EV characteristics. On this basis, ITGB1 levels were further compared between the two EV preparations by Western blot. With equal amounts of EV protein loaded per lane, ITGB1 was significantly reduced in shRUNX1 EVs relative to shNC EVs, whereas the canonical EV markers showed comparable expression levels between groups (Fig. 5A). To further determine whether this reduction was accompanied by altered EV number, we performed NTA on EVs isolated from equal numbers of shRUNX1 and shNC cells, and detected no significant difference in EV particle yield between groups (Supplementary Fig. 3B). These results indicate that RUNX1 modulates ITGB1 abundance in PDAC-derived EVs, with no obvious changes in overall EV number or the expression of core EV marker proteins. We further validated this regulatory relationship in vivo using ELISA, which confirmed markedly decreased ITGB1 abundance in plasma EVs isolated from mice with RUNX1-knockdown pancreatic orthotopic tumors compared with control tumor-bearing mice (Fig. 5B). Collectively, our in vitro and in vivo results support the regulatory role of RUNX1 in modulating ITGB1 abundance within PDAC-derived EVs.

To investigate whether EV ITGB1 levels were associated with PDAC-LM and patient clinical outcomes, we collected plasma samples from 38 PDAC patients (with complete clinical follow-up data) and 30 healthy individuals at Chongqing People’s Hospital. EVs were isolated from these plasma samples, with their size distribution and morphology characterized via NTA and TEM (Fig. 5C, D). Mass spectrometry differential analysis revealed significant ITGB1 upregulation in the plasma EVs from PDAC patients versus healthy controls (Fig. 5E, F and Supplementary Fig. 3C). GO (Gene Ontology) enrichment analysis revealed predominant enrichment of differentially expressed proteins in ECM organization, cell adhesion, integrin-mediated interactions, and acute inflammatory response (Fig. 5G). Meanwhile, Kyoto Encyclopedia of Genes and Genomes (KEGG) pathway analysis indicated their association with ECM-receptor interaction, focal adhesion, and PI3K-Akt/NF-κB signaling pathways (Fig. 5H). ELISA further validated this observation, revealing that ITGB1 levels in plasma EVs were not only significantly higher in PDAC patients overall but also particularly elevated in those with liver metastasis (Fig. 5I). Plasma EV ITGB1, quantified by ELISA, exhibited good diagnostic performance in differentiating PDAC patients from healthy controls (AUC = 0.8066; Fig. 5J) and in distinguishing PDAC patients with liver metastasis from those without (AUC = 0.7917; Fig. 5K). Importantly, Kaplan–Meier analysis of our cohort revealed that PDAC patients with high plasma EV ITGB1 levels exhibited significantly poorer OS (Fig. 5L). Collectively, these results not only confirm that RUNX1 regulates EV ITGB1 levels but also establish that EV ITGB1 is enriched in plasma EVs of PDAC patients with liver metastasis and serves as a potential diagnostic and prognostic indicator.

### ITGB1-enriched EVs derived from PDAC cells promote liver metastasis in vivo

**Fig. 6:**
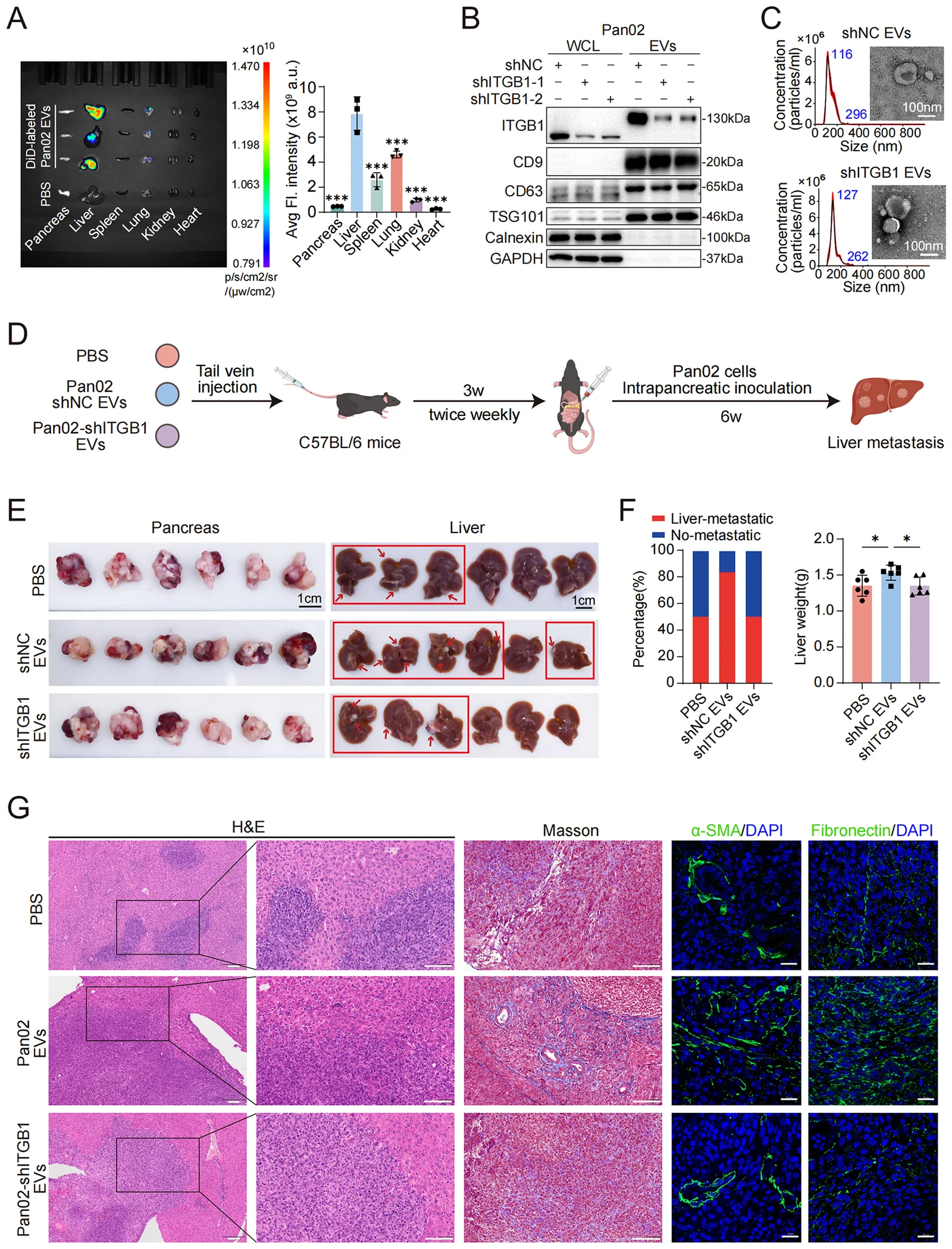
ITGB1-enriched EVs derived from PDAC cells promote liver metastasis in vivo. (**A**) Fluorescence intensity analysis showed the organotropism of DiD-labeled EVs secreted by PDAC cells 24 h after tail vein injection (*n* = 3). (**B**) Western blot validation of ITGB1 levels and canonical EV markers in EVs isolated from culture supernatants of shNC and shITGB1 Pan02 cells, with paired whole-cell lysates (WCL) loaded in parallel. (**C**) Characterization of EVs from Pan02 cells by TEM and NTA. (**D**) Schematic of the liver metastasis model established after EVs injection. (**E**) Pancreases and livers from mice sacrificed 6 weeks after orthotopic pancreatic injection of Pan02 cells (*n* = 6). (**F**) Liver metastatic rate and liver weight were measured. (**G**) H&E, Masson’s trichrome staining (Scale bar, 100 μm), and IF staining (Scale bar, 20 μm) for α-SMA and fibronectin in liver metastatic lesions. Data are presented as mean ± SD. Statistical analyses were performed using one-way ANOVA followed by Dunnett’s test. *P < 0.05, ***P < 0.001

To explore whether RUNX1 promotes PDAC-LM via ITGB1-enriched EVs, we first assessed the organotropism of EVs secreted by PDAC cells. DiD-labeled Pan02 cell-derived EVs were injected into mice via the tail vein. Fluorescence intensity analysis demonstrated that the liver exhibited higher accumulation of EVs compared with other organs (Fig. 6A). To verify the impact of ITGB1-enriched EVs secreted by PDAC cells on liver metastasis, EVs were isolated from the culture supernatants of ITGB1-knockdown and control Pan02 cells, with markedly reduced ITGB1 levels confirmed in the knockdown group (Fig. 6B). Both preparations displayed characteristic EV features, with the expected morphology, size distribution, and particle-to-protein ratio (Fig. 6C, Supplementary Table 5). These validated EVs were then used to pre-condition mice via tail vein injection prior to orthotopic implantation of Pan02 cells, establishing an EV-educated liver metastasis model (Fig. 6D). The results demonstrated that ITGB1-enriched EVs promoted liver metastasis, while ITGB1 knockdown reduced liver metastatic rate (Fig. 6E, F). No notable alteration was detected in the volume of pancreatic orthotopic tumors (Supplementary Fig. 4A). H&E staining further confirmed the presence of liver metastasis (Fig. 6G). Masson’s trichrome and IF staining of α-smooth muscle actin (α-SMA) and fibronectin demonstrated that treatment with ITGB1-enriched EVs increased fibrosis in liver metastatic lesions (Fig. 6G). Taken together, these findings suggest that ITGB1-enriched EVs function as critical promoters of PDAC-LM.

### ITGB1-enriched EVs derived from PDAC cells induce liver fibrotic pre-metastatic niche formation

**Fig. 7:**
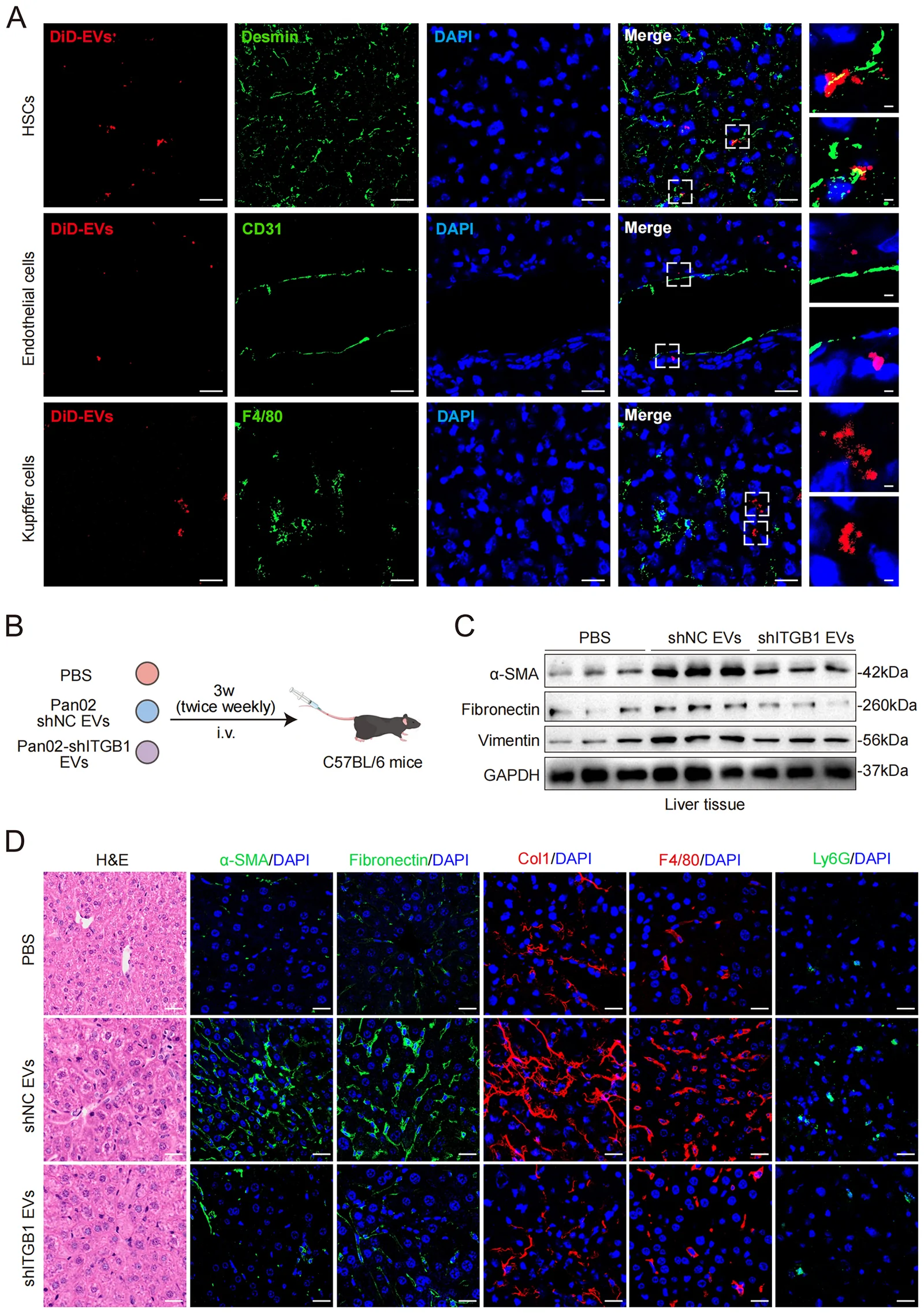
ITGB1-enriched EVs derived from PDAC cells induce liver fibrotic pre-metastatic niche formation. (**A**) IF analysis of tissue-resident stromal cells in mouse liver 24 h following tail vein injection of DiD-labeled EVs. Scale bar: 20 μm (main); 2 μm (insets) (*n* = 5). (**B**) Schematic of EVs tail vein injection model. (**C**) Western blot analysis of mouse liver after tail vein injection of PBS or EVs. (**D**) Representative H&E and IF images of α-SMA, fibronectin, Col1, F4/80, and Ly6G in the liver after injection of PBS or EVs. Scale bar, 20 μm

To investigate the mechanism by which ITGB1-enriched EVs promote liver metastasis, we performed IF staining on mouse livers following tail vein injection of DiD-labeled Pan02 cell–derived EVs. To delineate which hepatic cell population preferentially internalizes these EVs, dual immunofluorescence co-staining was carried out on parallel sections, pairing the DiD signal with Desmin, F4/80, or CD31 to mark hepatic stellate cells (HSCs), Kupffer cells, and liver sinusoidal endothelial cells, respectively. The proportion of DiD⁺ puncta co-localizing with each lineage marker was then quantified on a per-field basis. Among the three populations examined, Desmin⁺ HSCs accounted for the predominant share of EV uptake (58.8% ± 6.3%), markedly surpassing that of F4/80⁺ Kupffer cells (23.7% ± 3.0%) and CD31⁺ endothelial cells (10.0% ± 4.0%), with a highly significant difference across groups (Fig. 7A, Supplementary Fig. 4B). These findings identify HSCs as the principal cellular recipient of PDAC-derived EVs within the hepatic microenvironment—a cell type that serves as the major source of extracellular matrix (ECM) production and fibrous tissue deposition in the liver. Accordingly, we next examined ECM alterations in the liver following tail vein injection of PBS or EVs harboring different levels of ITGB1, administered twice weekly for three weeks (Fig. 7B). As expected, livers from mice pretreated with Pan02 cell-derived EVs exhibited increased ECM protein expression, whereas no significant increase in ECM deposition was observed in the ITGB1-knockdown EV group (Fig. 7C). Activation of HSCs accompanied by significantly increased deposition of α-SMA, type I collagen (Col1), and fibronectin in comparison to control mice was also confirmed by IF (Fig. 7D). Interestingly, livers from mice treated with Pan02 cell-derived EVs contained more Kupffer cells and infiltrating Ly6G^+^ myeloid cells than those from control mice (Fig. 7D). As previously reported, in addition to ECM deposition, myeloid cell accumulation has also been identified as a key feature of the PMN [31]. These findings indicate that ITGB1-enriched EVs secreted by PDAC cells promote fibrotic PMN formation in the liver, likely through activation of HSCs.

### ITGB1-enriched EVs activate HSCs via the NF-κB signaling pathway

**Fig. 8:**
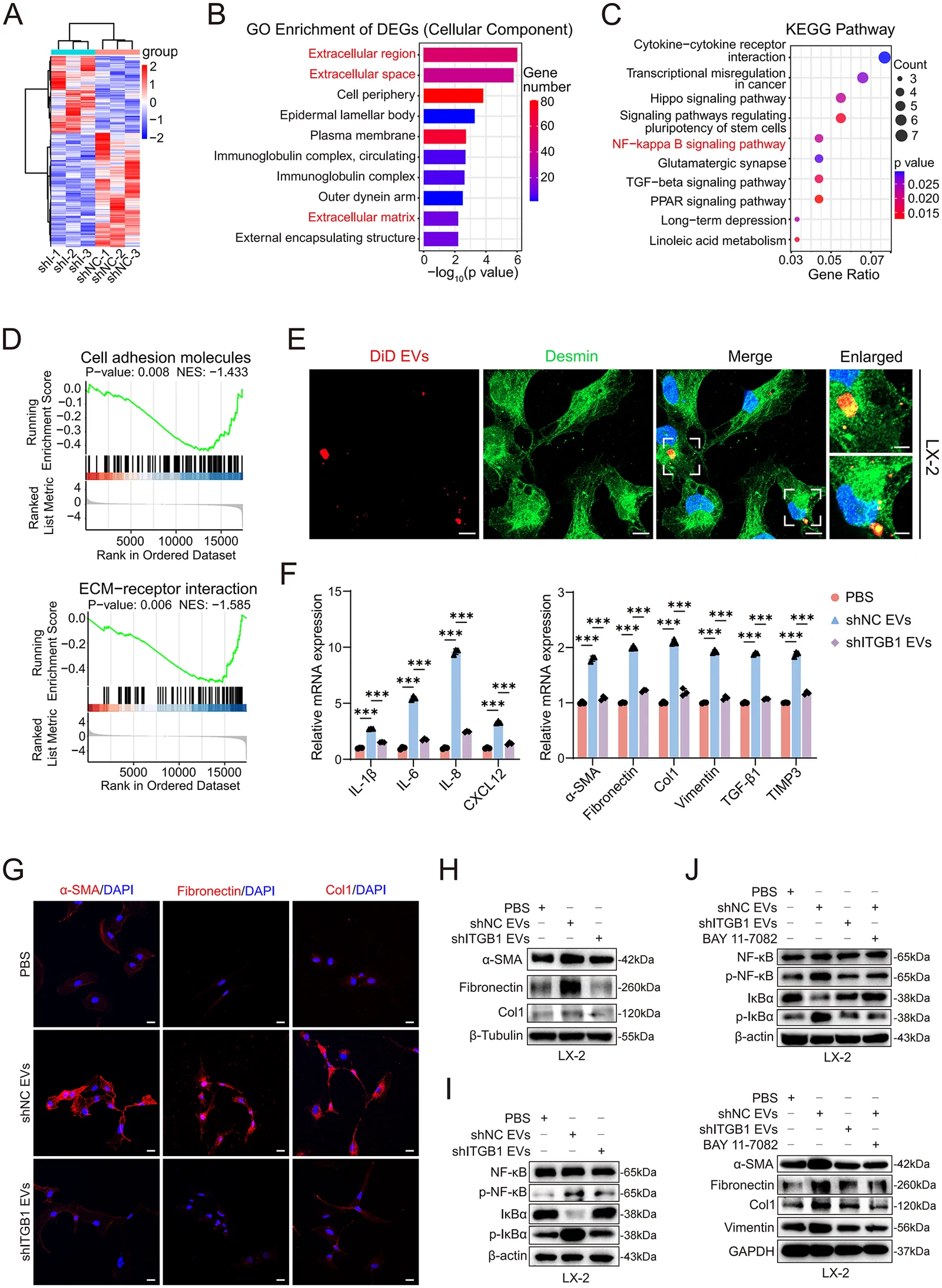
ITGB1-enriched EVs activate HSCs via NF-κB signaling pathway. (**A**) Heatmap of DEGs in mouse livers 3 weeks after injection with EVs derived from shITGB1-Pan02 or shNC-Pan02 cells (*n* = 3). (**B**-**C**) GO and KEGG pathway analysis of DEGs. (**D**) GSEA revealed a trend toward negative enrichment of the Cell Adhesion Molecules and ECM-Receptor Interaction pathways in the ITGB1-knockdown EV group. (**E**) Representative IF images showing internalization of DiD-labeled CFPAC-1-derived EVs by LX-2 cells. Scale bar: 10 μm (main); 4 μm (insets). (**F**) qRT-PCR detection of proinflammatory genes, chemokines, and fibrotic markers expression in LX-2 cells after 48 h co-incubation with PBS or CFPAC-1-derived EVs. (**G**-**H**) WB and IF analyses of α-SMA, fibronectin, and Col1 expression in LX-2 cells treated with PBS or CFPAC-1-derived EVs. Scale bar: 20 μm. (**I**) WB analysis of the NF-κB signaling pathway in LX-2 cells. (**J**) Western blot of NF-κB pathway components and fibrosis markers in LX-2 cells treated with PBS, shNC EVs, shITGB1 EVs, or shNC EVs plus BAY 11-7082. One-way ANOVA followed by Tukey’s post-hoc test was utilized for statistical analyses. ***P < 0.001

To delineate the mechanisms by which ITGB1-enriched EVs regulate HSC behavior, we compared the RNA-seq gene expression profiles of liver tissues from mice 3 weeks after injection with EVs derived from shITGB1-Pan02 or shNC-Pan02 cells (Fig. 8A). To identify key pathways and biological processes involved, we subjected the DEGs to GO and KEGG pathway analysis. In the GO cellular component category, the top enriched terms included extracellular space, extracellular region, and ECM (Fig. 8B)—consistent with our previous observation that PDAC cell-derived ITGB1-enriched EVs directly regulate ECM secretion. Furthermore, KEGG pathway analysis showed that the NF-κB signaling pathway was one of the most markedly impacted pathways in response to ITGB1-enriched EVs (Fig. 8C). Gene Set Enrichment Analysis (GSEA) revealed a trend toward negative enrichment of the Cell Adhesion Molecules pathway (normalized enrichment score [NES] = − 1.433, *P* < 0.01) and ECM-Receptor Interaction pathway (NES = − 1.585, *P* < 0.01) in the ITGB1-knockdown EV group (Fig. 8D).

To validate the effects of PDAC-derived ITGB1-enriched EVs on HSCs in vitro and dissect the underlying mechanisms, we used IF staining to confirm that DiD-labeled CFPAC-1 cell-derived EVs could be internalized by the human hepatic stellate cell line LX-2 (Fig. 8E). Consistent with our in vivo findings, co-incubating LX-2 cells with CFPAC-1 cell-derived EVs for 48 h led to the elevated expression of key proinflammatory genes/chemokines (IL-1β, IL-6, IL-8, CXCL12), fibrotic markers (TGF-β, α-SMA, fibronectin, Col1, vimentin, TIMP3)—effects that were abrogated by ITGB1 knockdown (Fig. 8F). Both IF and WB assays further confirmed that ITGB1-enriched EVs markedly induced LX-2 activation (Fig. 8G, H). Mechanistically, ITGB1-enriched EVs increased the expression of phosphorylated NF-κB, reduced IκBα levels, and thereby activated the NF-κB signaling pathway—whereas ITGB1 silencing exerted the opposite effect (Fig. 8I). To further confirm that NF-κB signaling functionally mediates the pro-fibrotic effects of ITGB1-enriched EVs on HSCs, we performed a rescue experiment using BAY 11-7082, a selective NF-κB pathway inhibitor. Pretreatment of HSCs with BAY 11-7082 effectively reversed the ITGB1-enriched EV-induced activation of the NF-κB pathway. Moreover, the upregulation of fibrosis-associated markers triggered by ITGB1-enriched EVs was significantly attenuated upon NF-κB inhibition (Fig. 8J). Taken together, these findings demonstrate that PDAC cell-derived ITGB1-enriched EVs promote HSC activation and fibrotic responses in vitro via the NF-κB signaling axis.

## Discussion

The clinical management of PDAC is severely hampered by its high propensity for liver metastasis, a process shaped by intricate reciprocal interactions between primary pancreatic cancer cells and the hepatic microenvironment [3, 32]. In this study, we identified RUNX1 as a key regulator of PDAC liver metastasis and characterized a novel RUNX1–EV–ITGB1 transmission regulatory axis, through which PDAC cells remodel the hepatic premetastatic niche to facilitate metastatic outgrowth. By linking a primary-tumor transcription factor to EV cargo composition and downstream stromal activation, our work integrates tumor-intrinsic regulation and pre-metastatic niche biology within a single coherent framework. These findings provide mechanistic insight into PDAC liver metastasis and offer prognostic and diagnostic biomarkers as well as therapeutic candidates for this lethal disease.

RUNX1 has been well characterized as a pivotal oncogene in hematological malignancies [33, 34], while its function in solid tumors remains controversial. It has been documented to play a central regulatory role in driving key cancer progression processes, including proliferation, angiogenesis, chemoresistance to anticancer drugs, cancer stemness, and cancer cell metastasis in some tumors such as colorectal cancer, hepatocellular carcinoma, and renal cell carcinoma [35–37]. In particular, in colorectal cancer, RUNX1 has been demonstrated by multiple studies to promote liver metastasis [37, 38]. However, in breast cancer and gastric cancer, RUNX1 has been found to exert a tumor-suppressive role [39, 40]. In PDAC, prior studies indicated that RUNX1 is upregulated in PDAC tissues and may promote PDAC progression through transcriptional modulation of C-FOS [26, 41], but its role in organ-specific metastasis (especially liver) remained elusive. By integrating transcriptomic profiling of a treatment-naive PDAC cohort with orthotopic murine models, our work links RUNX1 to liver metastasis and provides functional evidence supporting its role. RUNX1 increased metastatic burden and exacerbated hepatic fibrosis—a hallmark of the desmoplastic microenvironment that fuels PDAC progression—identifying it as a relevant contributor to PDAC liver metastasis through both tumor cell-intrinsic aggressiveness and stromal remodeling.

EVs serve as critical mediators of intercellular communication by packaging and delivering functional biomolecules between cells. Emerging evidence indicates that EVs function as a pivotal mediator in driving cancer progression, encompassing key events like PMN formation and metastasis, by altering ligand-receptor interactions and cargo release in recipient cells [42]. Integrins, one of the most commonly expressed proteins on the surface of EVs, have been shown to be transported via EVs to specific tissues, where they interact with cells or the ECM, thereby contributing to PMN formation and tumor metastasis [29, 30]. In prostate cancer, higher levels of ITGA3 and ITGB1 were found in urinary EVs of metastatic patients than in those with benign prostate hyperplasia or non-metastatic prostate cancer, and inhibiting these EV integrins prevented the migration and invasion of non-cancerous prostate epithelial cells [43]. In breast cancer, ITGB1 originating from breast cancer cells may be shuttled to recipient cells via EVs to enhance migration and metastasis [44]. While in adenoid cystic carcinoma, EV integrin α2β1 from CAFs can also induce metastasis by acting on recipient cells [45]. However, the specific role of EV-associated ITGB1 in PDAC liver metastasis has remained largely unexplored. Our study identified EV ITGB1 as a clinical correlate of PDAC-LM and provided evidence supporting its role in the activation of HSCs and PDAC-LM. Moreover, by establishing RUNX1 as a transcriptional regulator of ITGB1, we connect upstream transcriptional control with EV cargo composition, characterizing the RUNX1–ITGB1 axis as a novel mechanism contributing to PDAC liver metastasis and offering new insight into PMN biology. The mechanism by which ITGB1 is incorporated into PDAC-derived EVs, however, remains to be defined. Although our data demonstrate consistent ITGB1 enrichment in EVs relative to whole-cell lysates, the precise sorting determinants were not experimentally dissected here. Plausible mechanisms supported by existing literature include galectin-3-mediated integrin export into EVs [46], tetraspanin-enriched microdomain-based cargo organization [47], Rab21-governed ITGB1 endosomal trafficking [48], and perturbation of endosomal recycling that redirects ITGB1 toward EV secretion [49]. Whether one or several of these mechanisms operate in PDAC, and whether RUNX1 itself influences EV cargo composition beyond its transcriptional regulation of ITGB1, represent important directions for future investigation.

From a translational standpoint, plasma EV ITGB1 emerges as a clinically actionable, non-invasive biomarker with favorable diagnostic performance for identifying PDAC-LM patients, supporting its utility in risk stratification, prognostic monitoring, and as a candidate therapeutic target. Non-invasive blood-based biomarkers have gained increasing traction for risk stratification and prognostic surveillance in hepatobiliary and pancreatic malignancies, with circulating nucleic acids, soluble proteins, and EV-associated cargoes emerging as complementary readouts of disease state [50–52]. Plasma EV ITGB1 represents a mechanistically anchored addition to this evolving biomarker toolkit, with the distinct advantage of being directly linked to the fibrotic niche-forming process underlying PDAC liver metastasis.

The generation of hepatic PMN relies critically on fibrosis triggered by HSC activation, and elevated severity of liver fibrosis markedly raises the risk of PDAC-LM [53–55]. In line with this mechanism, our in vitro studies demonstrated that HSC activation was markedly enhanced following EV internalization, as evidenced by upregulation of ECM proteins, inflammatory factors, and chemokines. This regulatory effect was verified in vivo by assessing liver fibrosis severity in murine models following tail vein administration of ITGB1-enriched EVs. Consistent with this observation, previous studies have confirmed that EVs derived from colorectal cancer are capable of being internalized by HSCs, ultimately promoting the formation of the hepatic PMN [18, 56]. Additionally, our IF staining observations revealed that ITGB1-enriched EVs not only contributed to fibrotic microenvironment formation but also resulted in a notable increase in hepatic infiltration of Kupffer cells and LY6G⁺ myeloid cells—cell populations widely recognized as contributors to the immunosuppressive milieu and PMN formation [31], though the specific regulatory roles of these infiltrating cells in our PDAC model await further mechanistic exploration. Such observations are broadly consistent with prior reports linking the expansion of immunosuppressive myeloid populations to chronic hepatic injury and tumor progression [57], underscoring the relevance of myeloid-driven immune permissiveness to the hepatic pre-metastatic landscape. Consistently, our GSEA pathway analysis specifically showed that ECM-receptor interaction and Cell Adhesion Molecules pathways were downregulated in livers treated with EVs from ITGB1-knockdown Pan02 cells compared with those treated with EVs from negative control Pan02 cells. This finding not only reflects impaired ECM-related crosstalk and attenuated adhesive interactions (core processes shaping the fibrotic microenvironment and mediating cell recruitment and retention [58, 59]) but also supports the role of EV ITGB1 in regulating these pathways and sustaining the pro-metastatic hepatic microenvironment.

Notably, we identified NF-κB pathway activation as a downstream effector of EV ITGB1 in HSCs, providing mechanistic insight into how EV-derived ITGB1 promotes HSC activation. Given that NF-κB is a well-characterized regulator of hepatic inflammatory and fibrotic responses [60, 61], these findings collectively highlight a functional RUNX1–ITGB1–NF-κB axis—a potentially promising therapeutic target to abrogate hepatic PMN formation and thereby prevent or delay PDAC liver metastasis.

Several limitations of this study should be acknowledged. First, the sample size of our RNA-sequencing cohort was relatively limited, due to the rarity of surgically resected specimens from treatment-naive PDAC patients with synchronous liver metastasis. Larger multicenter cohorts are therefore required to further validate and consolidate our transcriptomic findings. Second, although hepatic infiltration of Kupffer cells and LY6G⁺ myeloid cells was observed following exposure to ITGB1-enriched EVs, the specific functional contributions of each immune subset to PMN formation warrant further investigation. Finally, while EV ITGB1 demonstrated favorable diagnostic efficacy in our cohort, prospective multicenter validation is required before clinical implementation as a routine biomarker.

## Conclusions

**Fig. 9:**
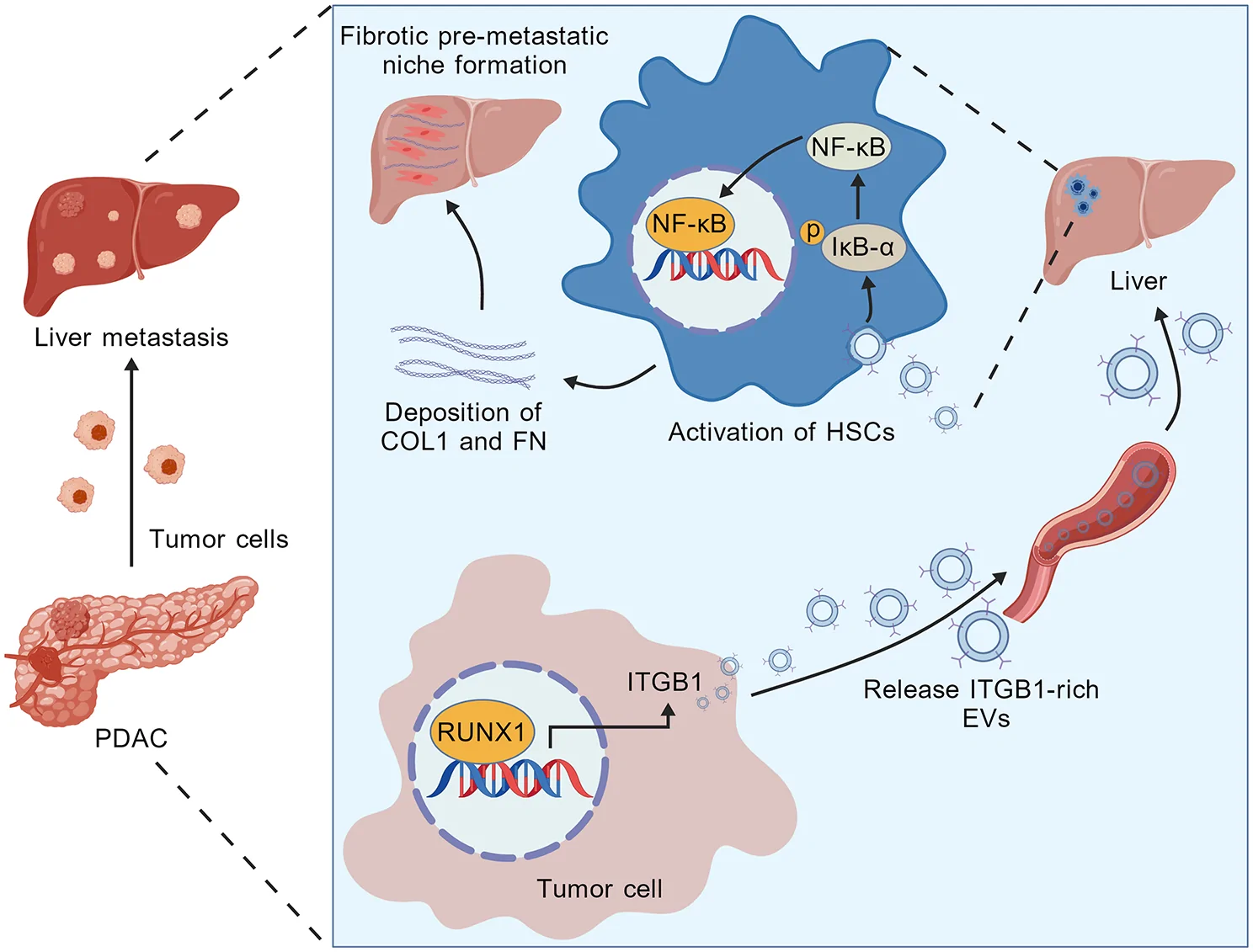
The schematic diagram of RUNX1-mediated ITGB1-enriched EVs promoting PDAC liver metastasis. In primary PDAC, RUNX1 upregulates ITGB1 transcription, thereby increasing ITGB1 levels in cancer cell-derived EVs. These ITGB1-enriched EVs are internalized by HSCs and activate the NF-κB signaling pathway to drive HSC activation, consequently promoting the formation of a fibrotic PMN in the liver and ultimately facilitating PDAC-LM

In summary, this study identifies a previously unrecognized RUNX1–ITGB1–NF-κB regulatory axis that contributes to fibrotic hepatic PMN formation and thereby promotes PDAC liver metastasis. Within primary PDAC cells, RUNX1 transcriptionally upregulates ITGB1, which is subsequently enriched in tumor-derived EVs and internalized by HSCs, where it activates NF-κB signaling and promotes stromal remodeling that may facilitate metastatic colonization (Fig. 9). By integrating primary-tumor transcriptional regulation, EV cargo composition, and stromal activation within a single coherent framework, our findings advance the mechanistic understanding of PDAC liver metastasis and characterize plasma EV ITGB1 as a clinically translatable, non-invasive biomarker for risk stratification and prognostic prediction. From a therapeutic perspective, the RUNX1–ITGB1–NF-κB axis presents multiple druggable nodes that could be exploited to prevent or delay hepatic metastatic progression. Future investigations should prioritize prospective multicenter validation of EV ITGB1 as a biomarker and advance the preclinical development of axis-targeting agents. Collectively, these efforts will help translate the present mechanistic insights into improved early detection, refined risk stratification, and targeted intervention for PDAC patients confronting the threat of liver metastasis.

## Supplementary Information

Below is the link to the electronic supplementary material.

Supplementary Material 1

Supplementary Material 2

Supplementary Material 3

## Data Availability

Data are available from the corresponding author as required.

## Abbreviations

LM: Liver metastasis
PDAC: Pancreatic ductal adenocarcinoma
PMN: Pre-metastatic niche
RUNX1: Runt-related transcription factor 1
ITGB1: Integrin beta 1
EVs: Extracellular vesicles
ECM: Extracellular matrix
TCGA: The Cancer Genome Atlas
GTEx: Genotype-Tissue Expression
FBS: Fetal bovine serum
RT: Room temperature
shRNA: Short hairpin RNA
TEM: Transmission electron microscopy
NTA: Nanosight particle tracking analysis
ELISA: Enzyme-linked immunosorbent assay
DFS: Disease-free survival
OS: Overall survival
GO: Gene Ontology
KEGG: Kyoto Encyclopedia of Genes and Genomes
ROC: Receiver operating characteristic
AUC: Area under the ROC curve
HSC: Hepatic stellate cell
α-SMA: α-smooth muscle actin
Col1: Type I collagen
DEGs: Differentially expressed genes
GSEA: Gene Set Enrichment Analysis
