# Supplementary material for: RUNX1-regulated ITGB1-enriched extracellular vesicles drive pancreatic cancer liver metastasis via fibrotic pre-metastatic niche formation
Source: J Transl Med. 2026 Jun 22;24:1142. doi: 10.1186/s12967-026-08521-3 (PMC13548723; doi:10.1186/s12967-026-08521-3)

Figure 2A

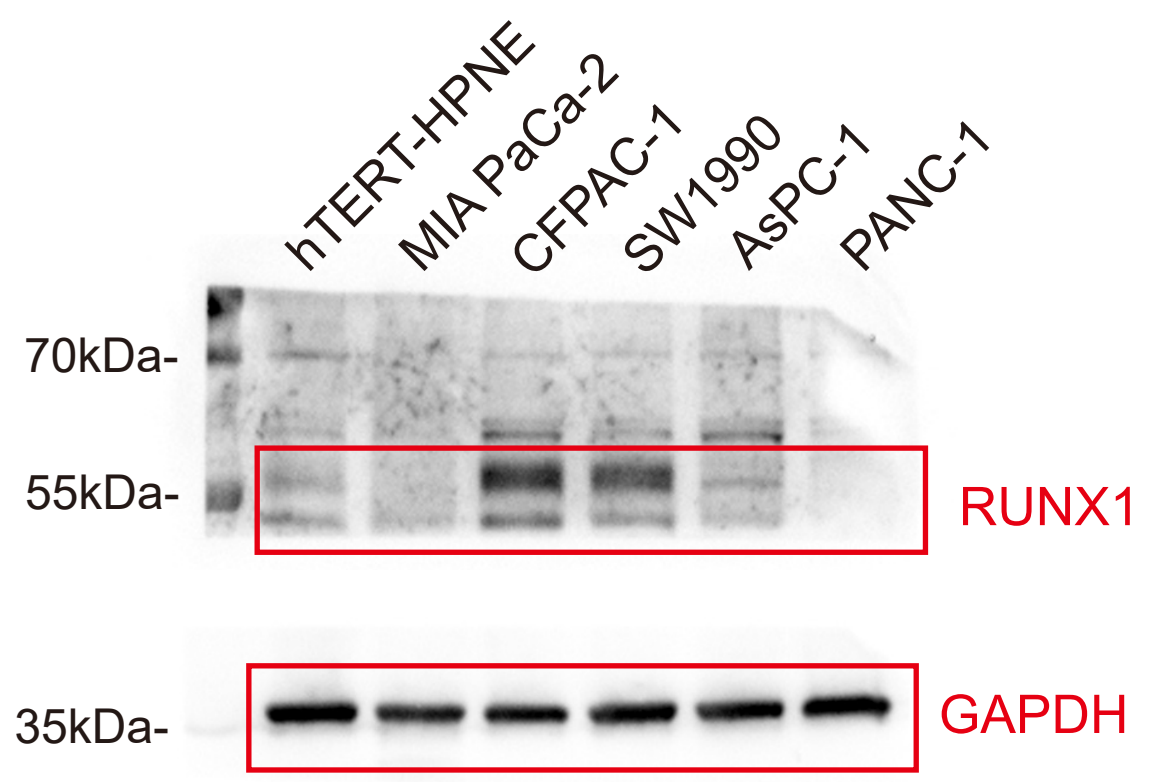

Figure 2B

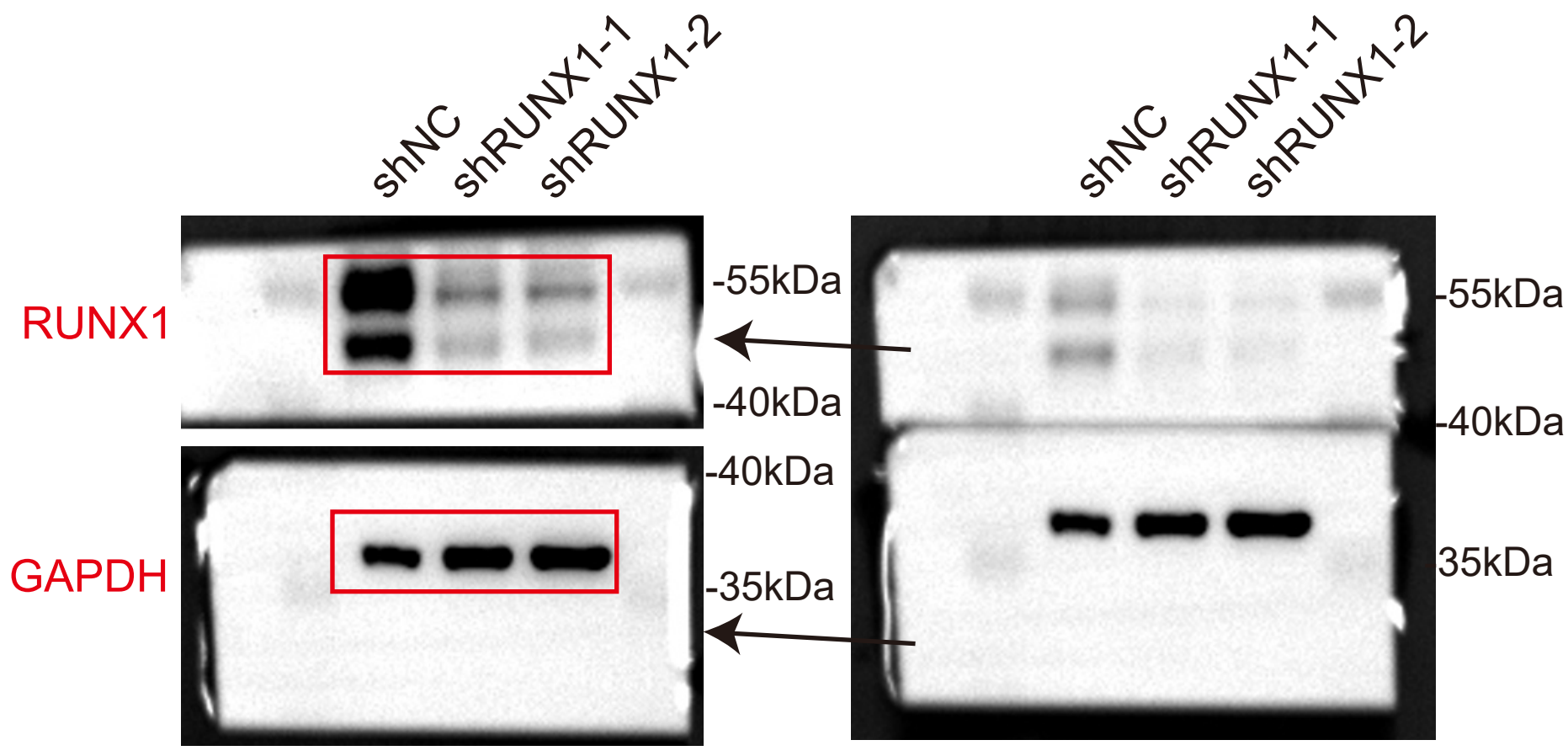

Figure 2C

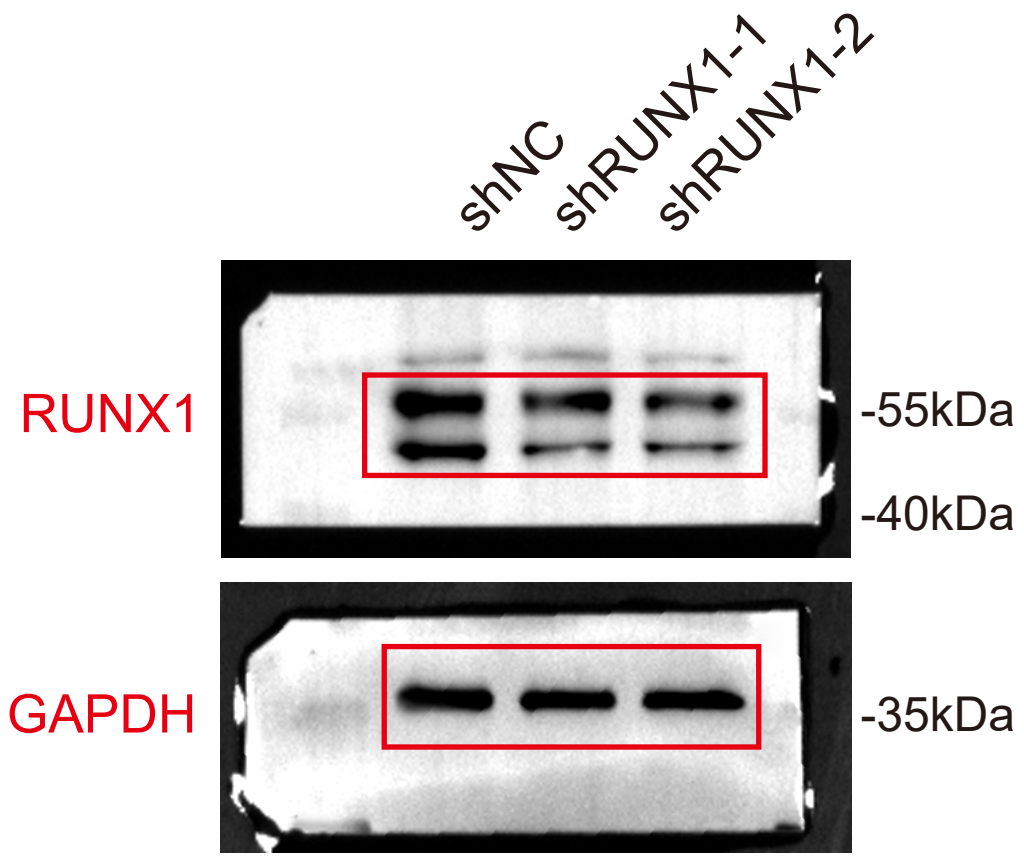

Figure 3H

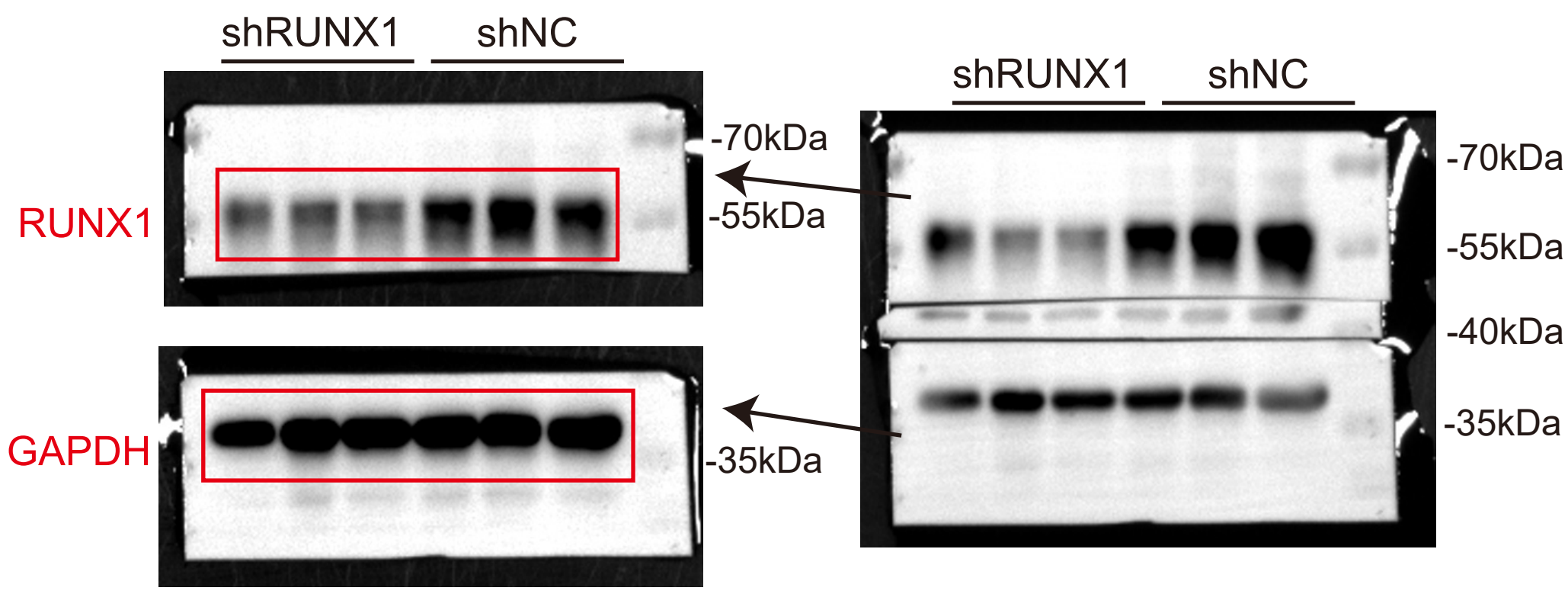

Figure 4E

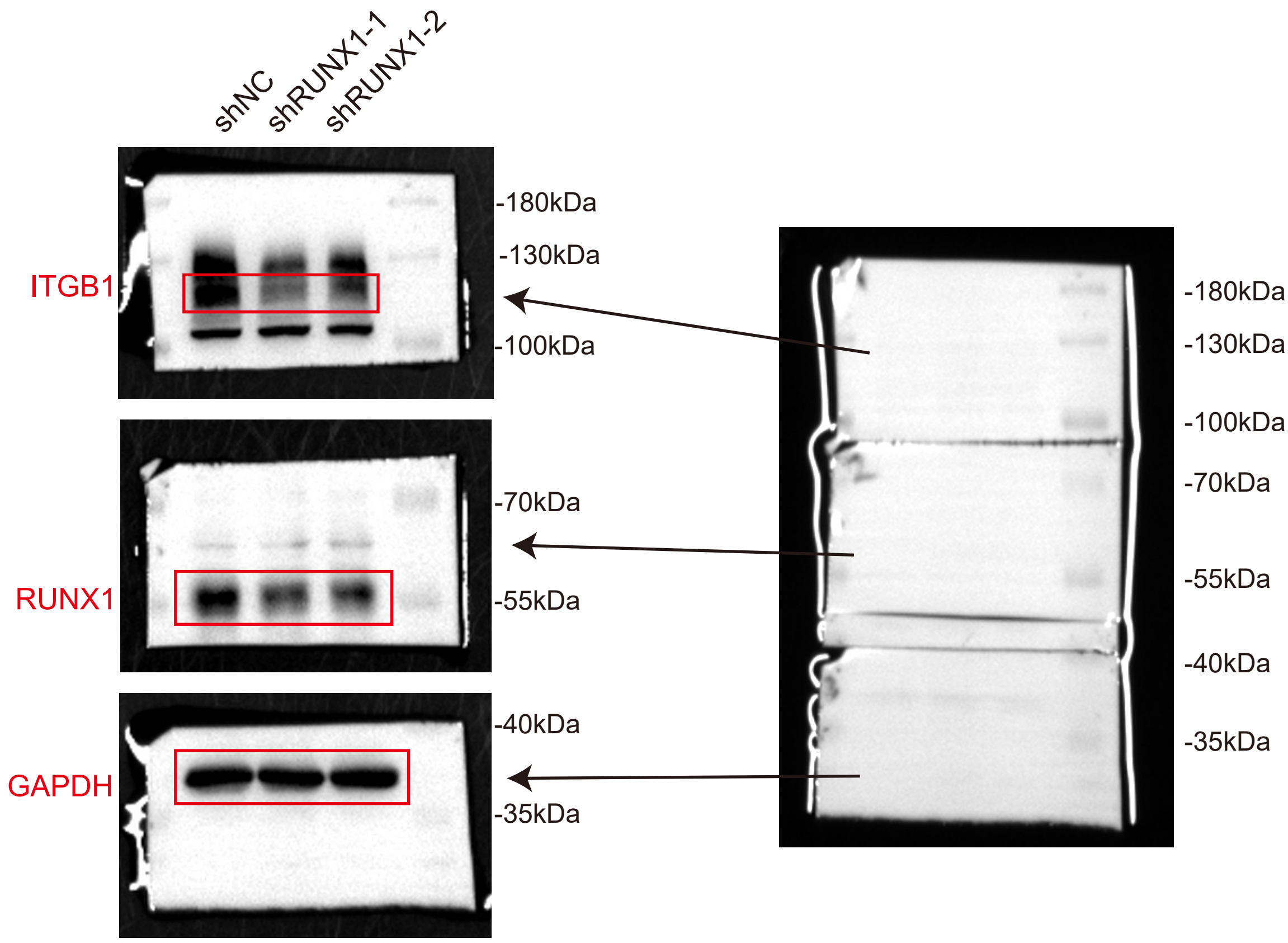

Figure 4F

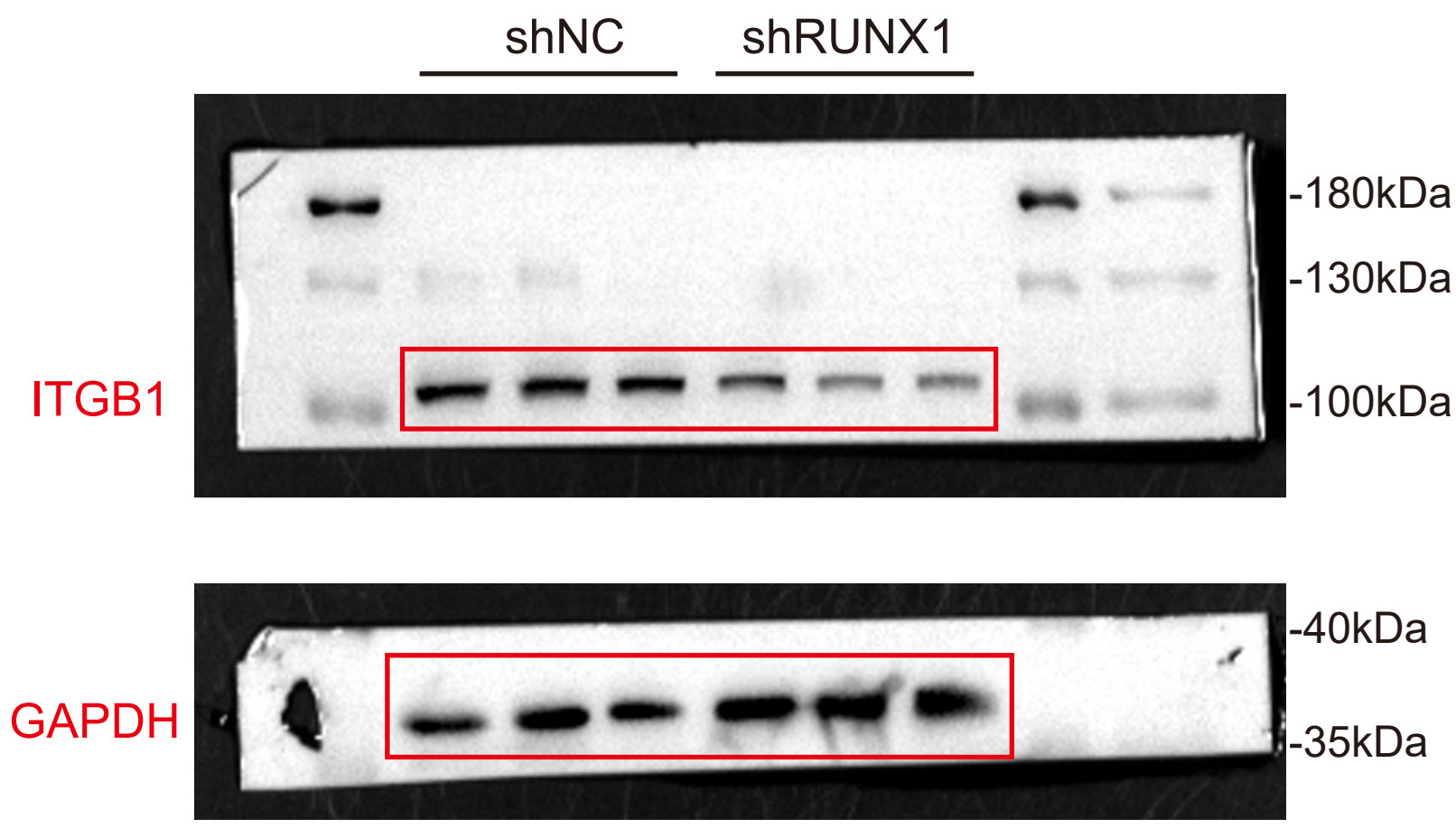

Figure 5A

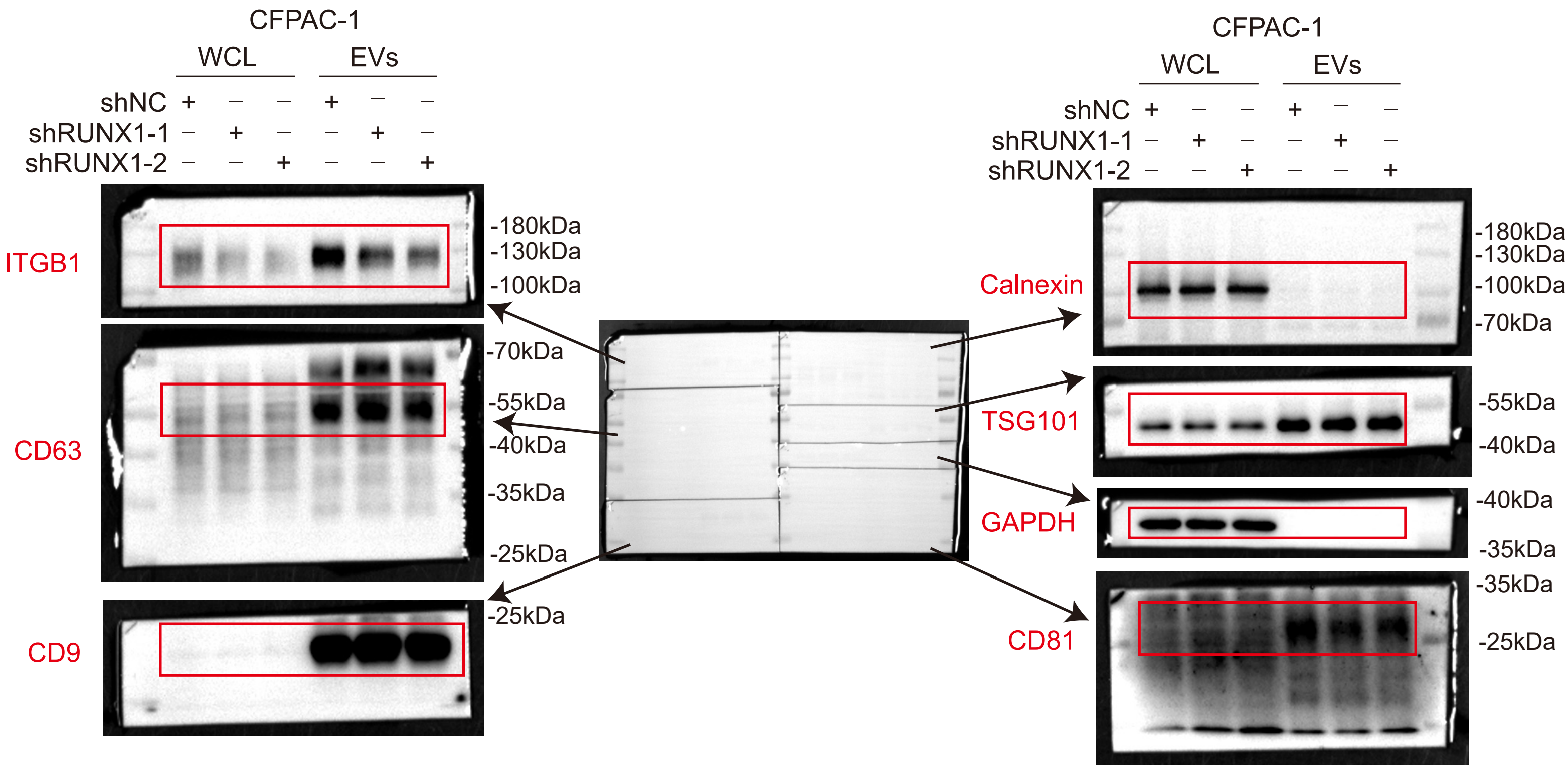

Figure 6B

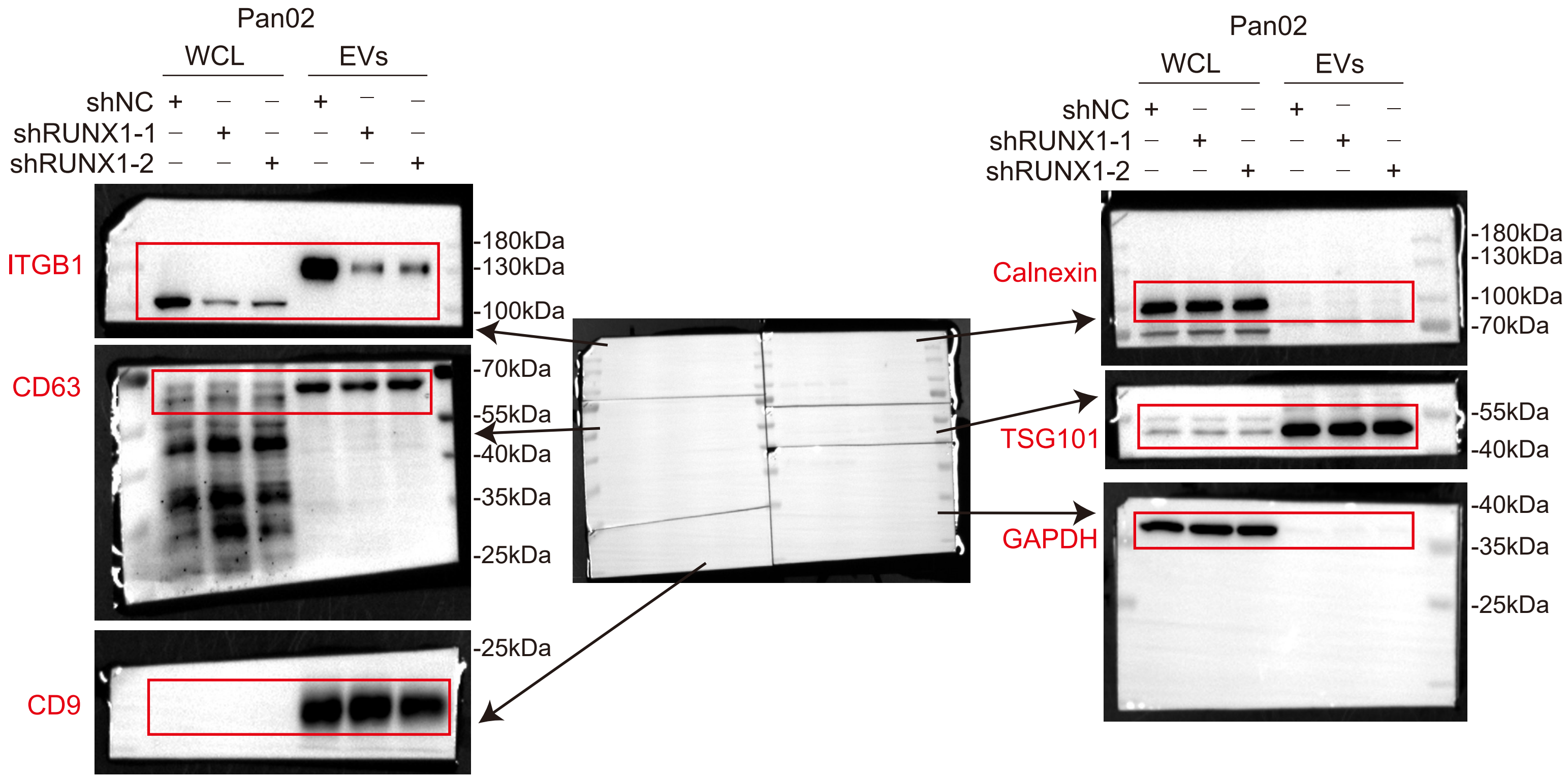

Figure 7C

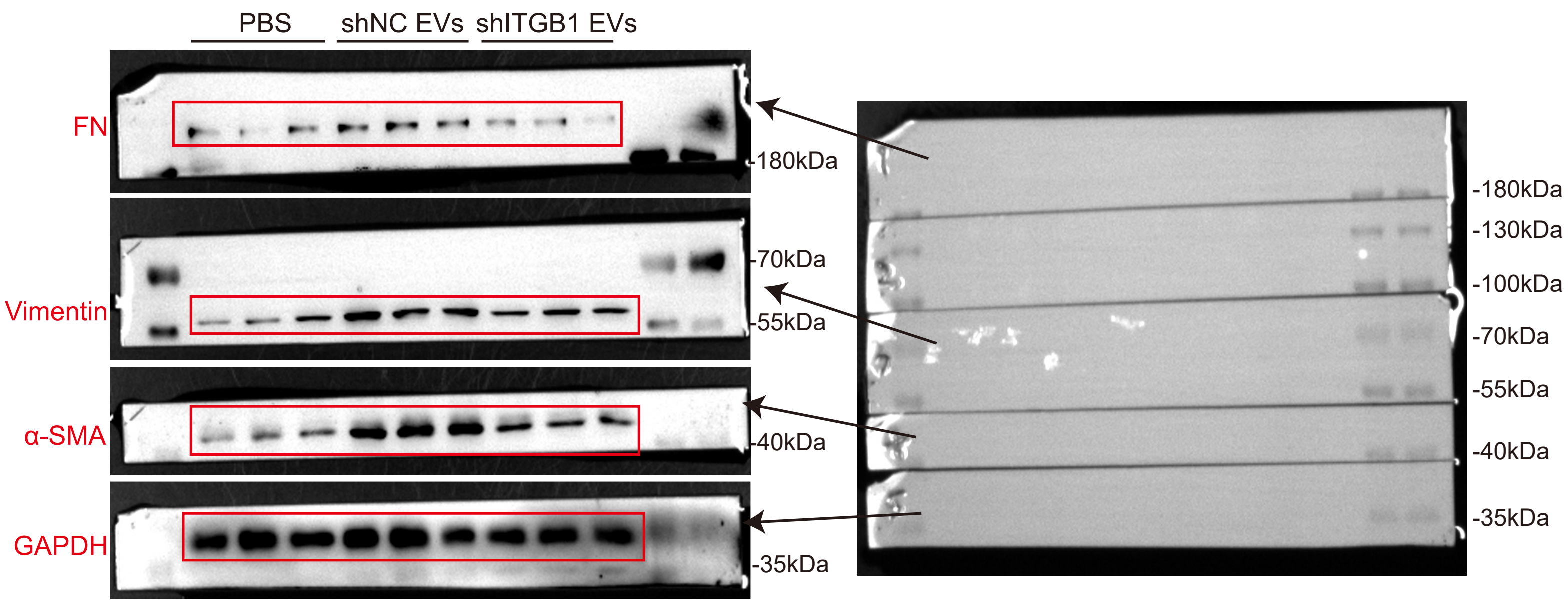

Figure 8H

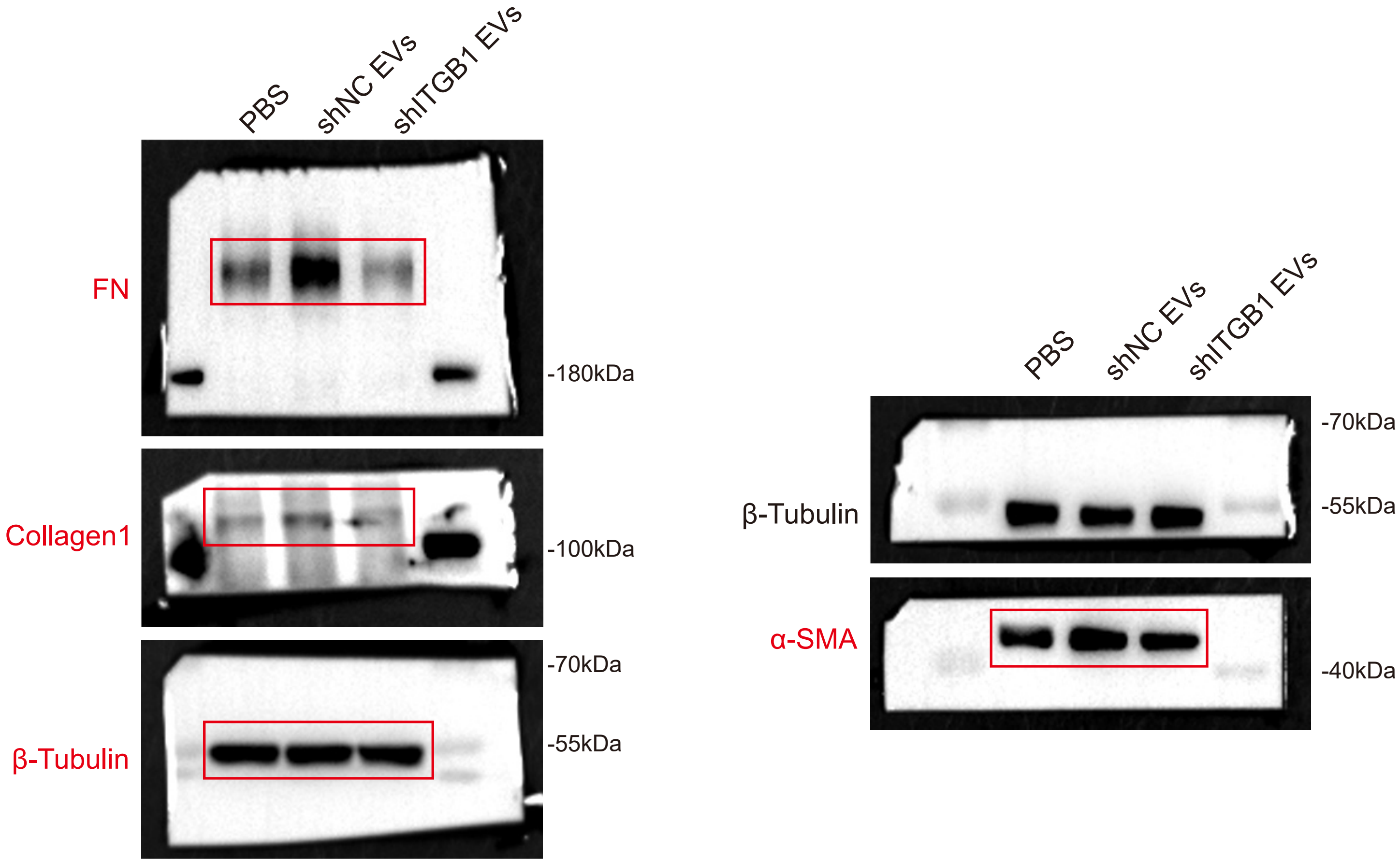

Figure 8I

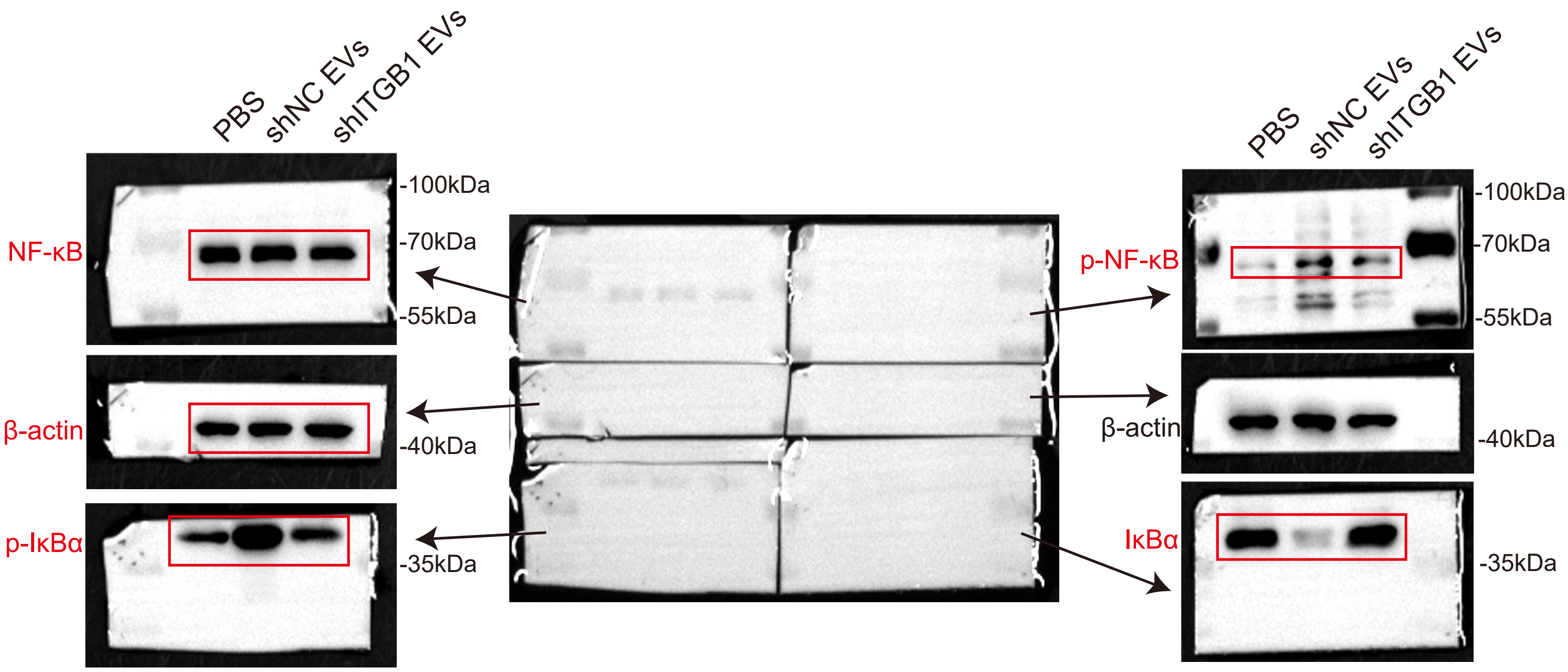

Figure 8J

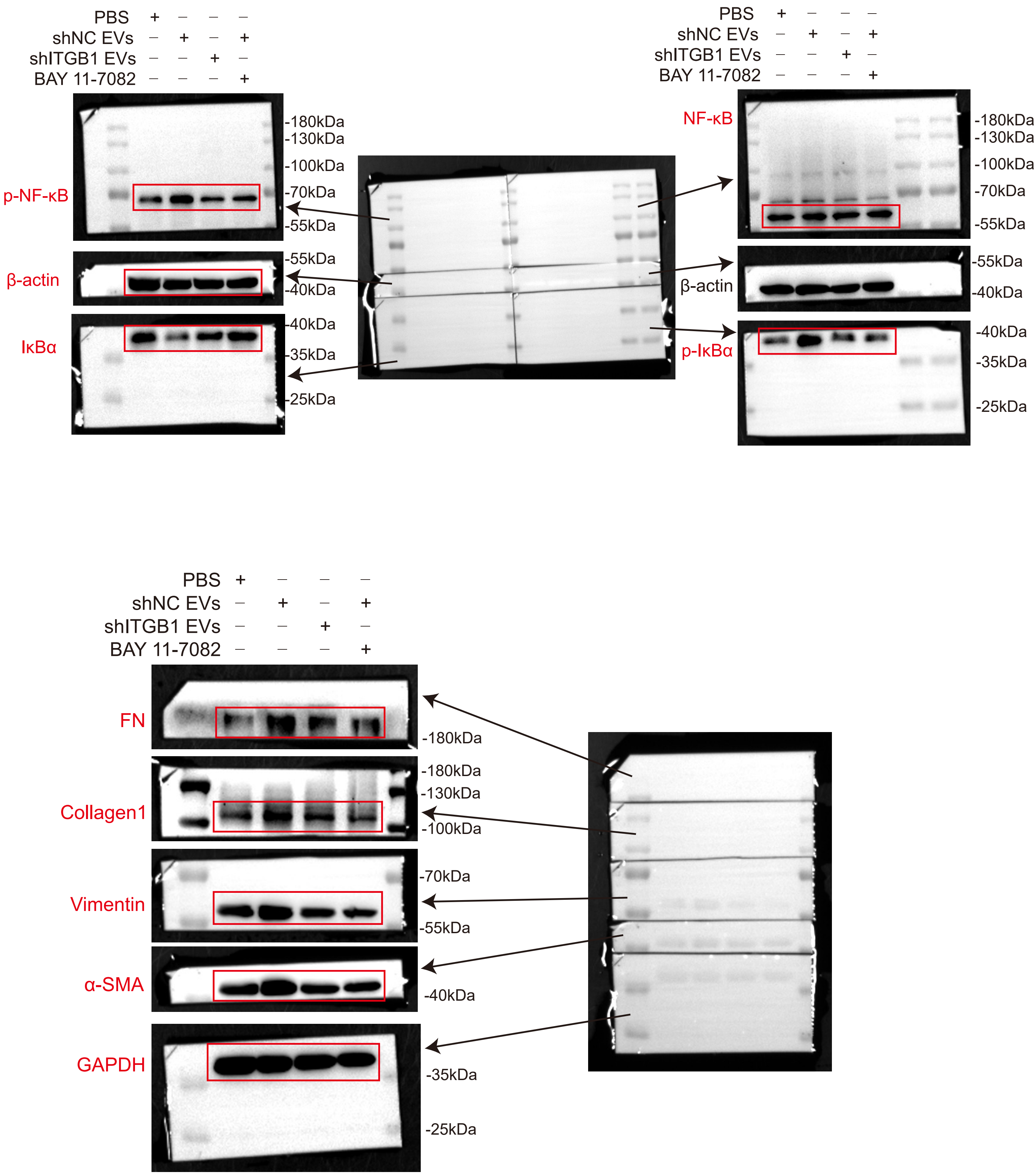
PBS + − − −
shNC EVs − + − +
shITGB1 EVs − − + −
BAY 11-7082 − − − +
p-NF-κB
β-actin
IκBα
-180kDa
-130kDa
-100kDa
-70kDa
-55kDa
-55kDa
-40kDa
-40kDa
-35kDa
-25kDa
PBS + − − −
shNC EVs − + − +
shITGB1 EVs − − + −
BAY 11-7082 − − − +
NF-κB
β-actin
p-IκBα
-180kDa
-130kDa
-100kDa
-70kDa
-55kDa
-55kDa
-40kDa
-40kDa
-35kDa
-25kDa
PBS + − − −
shNC EVs − + − +
shITGB1 EVs − − + −
BAY 11-7082 − − − +
FN
Collagen1
Vimentin
α-SMA
GAPDH
-180kDa
-180kDa
-130kDa
-100kDa
-70kDa
-55kDa
-40kDa
-35kDa
-25kDa

Supplementary Figure2A

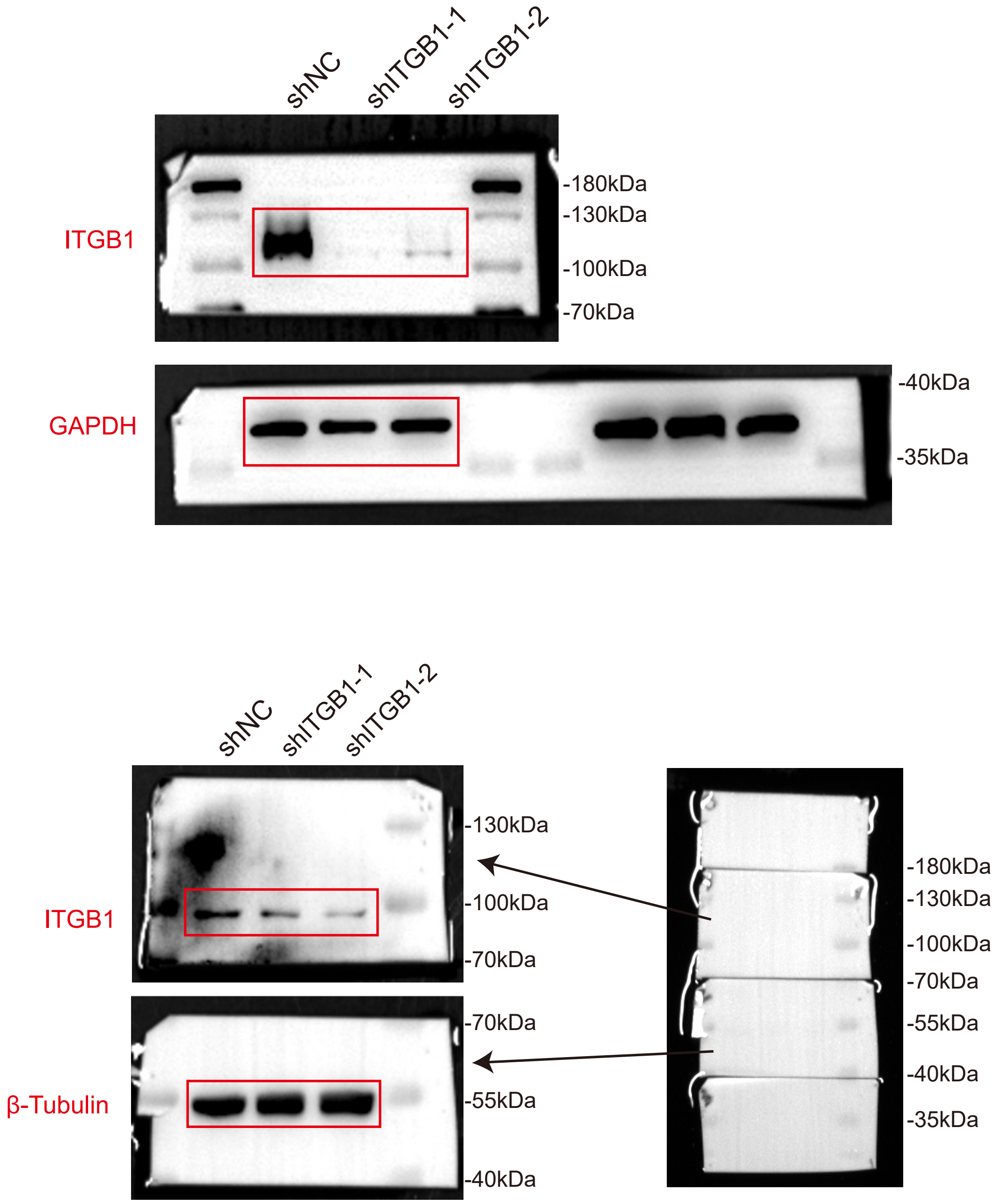

Supplement: Supplementary file 1 [file 12967_2026_8521_MOESM1_ESM.pdf]
