# Supplementary material for: RUNX1-regulated ITGB1-enriched extracellular vesicles drive pancreatic cancer liver metastasis via fibrotic pre-metastatic niche formation
Source: J Transl Med. 2026 Jun 22;24:1142. doi: 10.1186/s12967-026-08521-3 (PMC13548723; doi:10.1186/s12967-026-08521-3)
Supplement: Supplementary file 2 [file 12967_2026_8521_MOESM2_ESM.docx]

**Supplementary Fig. 1** **ITGB1 is a functional downstream target of RUNX1 in PDAC.** (A) Potential target genes of RUNX1 identified by TF-Target Finder. (B) Correlation of RUNX1 and ITGB1 expression levels across multiple cancer types and normal tissues. (C) ITGB1 expression in PDAC tissues versus normal pancreatic tissues.

**Supplementary Fig. 2 ITGB1 promotes PDAC cell proliferation, migration, and invasion in vitro.** (A) Validation of ITGB1 knockdown in CFPAC-1 and Pan02 cells by qRT-PCR and WB. (B) CCK-8 assay illustrating the proliferative capacity of ITGB1-knockdown Pan02 cells. (C) Wound-healing assay assessing cell migration. Scale bar, 500 μm. (D) Transwell assays showing the invasive capacity of Pan02 cells. Scale bar, 200 μm. Data are presented as mean ± SD. Statistical analyses were performed using unpaired Student’s t-test and one-way ANOVA followed by Dunnett’s test. ***P* < 0.01, ****P* < 0.001.

**Supplementary Fig. 3 Identification of EVs from CFPAC-1 cells and mass spectrometry analysis of plasma EVs.** (A) Characterization of EVs from CFPAC-1 cells by TEM and NTA. (B) EV particle yield from equal numbers of shNC and shRUNX1 CFPAC-1 cells, quantified by NTA (n = 3). (C) The heat map shows the differentially expressed proteins in the plasma EVs from PDAC patients versus healthy controls. Data are presented as mean ± SD. Statistical analyses were performed using unpaired Student’s t-test. ns, not significant (*P* ≥ 0.05).

**Supplementary Fig. 4 Additional in vivo characterization of EV-induced effects on the pancreatic tumor and hepatic microenvironment.** (A) Orthotopic tumor volume of mice pretreated with EVs containing different levels of ITGB1 or PBS followed by orthotopic injection of Pan02 cells (n = 6). (B) Quantification of DiD⁺ EV co-localization with Desmin⁺ HSCs, F4/80⁺ Kupffer cells, and CD31⁺ endothelial cells in mouse livers Data represent the percentage of DiD⁺ puncta co-localizing with each lineage marker per field (n = 5). Data are presented as mean ± SD. one-way ANOVA with Dunnett’s test; ****P* < 0.001, ns, not significant (*P* ≥ 0.05).
