# Supplementary material for: RUNX1-regulated ITGB1-enriched extracellular vesicles drive pancreatic cancer liver metastasis via fibrotic pre-metastatic niche formation
Source: J Transl Med. 2026 Jun 22;24:1142. doi: 10.1186/s12967-026-08521-3 (PMC13548723; doi:10.1186/s12967-026-08521-3)
Supplement: Supplementary file 3 [file 12967_2026_8521_MOESM3_ESM.docx]

**Supplementary Table 1.** Primers for qRT-PCR.

| **Species** | **Gene name** | **Fw primer 5’-3’** | **Rev primer 5’-3’** |
| --- | --- | --- | --- |
| Human | RUNX1 | TGAGCTGAGAAATGCTACCGC | ACTTCGACCGACAAACCTGAG |
| Human | ITGB1 | CCTACTTCTGCACGATGTGATG | CCTTTGCTACGGTTGGTTACATT |
| Human | GAPDH | GGAGCGAGATCCCTCCAAAAT | GGCTGTTGTCATACTTCTCATGG |
| Human | 18S | CGGCTACCACATCCAAGGAA | GCTGGAATTACCGCGGCT |
| Human | IL-6 | ACTCACCTCTTCAGAACGAATTG | CCATCTTTGGAAGGTTCAGGTTG |
| Human | IL-8 | TTTTGCCAAGGAGTGCTAAAGA | AACCCTCTGCACCCAGTTTTC |
| Human | IL-1β | ATGATGGCTTATTACAGTGGCAA | GTCGGAGATTCGTAGCTGGA |
| Human | CXCL12 | ATTCTCAACACTCCAAACTGTGC | ACTTTAGCTTCGGGTCAATGC |
| Human | α-SMA | GGCAAGTGATCACCATCGGA | GTGGTTTCATGGATGCCAGC |
| Human | TGF-β1 | GAGAAGCGGTACCTGAACCC | TGAACCCGTTGATGTCCACT |
| Human | fibronectin | CGGTGGCTGTCAGTCAAAG | AAACCTCGGCTTCCTCCATAA |
| Human | COL1 | CATAAAGGGTCACCGTGGCT | AGTCCATCTTTGCCAGGAGC |
| Human | vimentin | GACGCCATCAACACCGAGTT | CTTTGTCGTTGGTTAGCTGGT |
| Human | TIMP3 | CATGTGCAGTACATCCATACGG | CATCATAGACGCGACCTGTCA |
| Murine | RUNX1 | GATGGCACTCTGGTCACCG | GCCGCTCGGAAAAGGACAA |
| Murine | ITGB1 | ATGCCAAATCTTGCGGAGAAT | TTTGCTGCGATTGGTGACATT |
| Murine | GAPDH | AGGTCGGTGTGAACGGATTTG | TGTAGACCATGTAGTTGAGGTCA |

**Supplementary Table 2.** Antibodies utilised in this study.

| **Name** | **Product ID** | **Company** | **Dilution** | **Method** |
| --- | --- | --- | --- | --- |
| RUNX1 | ab240639 | Abcam | 1:1000 | WB |
| GAPDH | ab181602 | Abcam | 1:2000 | WB |
| ITGB1 | 12594-1-AP | Proteintech | 1:2000 | WB |
| CD9 | ab236630 | Abcam | 1:1000 | WB |
| CD81 | ab79559 | Abcam | 1:1000 | WB |
| Calnexin | 10427-2-AP | Proteintech | 1:1000 | WB |
| CD63 | ab134045 | Abcam | 1:1000 | WB |
| TSG101 | ab125011 | Abcam | 1:1000 | WB |
| beta Tubulin | ab6046 | Abcam | 1:2000 | WB |
| α-SMA | ab124964 | Abcam | 1:2000 | WB |
| fibronectin | ab268020 | Abcam | 1:1000 | WB |
| vimentin | T55134 | Abmart | 1:1000 | WB |
| COL1 | 1310-01 | SouthernBiotech | 1:1000 | WB |
| p-NF-κB | TP56372 | Abmart | 1:1000 | WB |
| NF-κB | T55034 | Abmart | 1:1000 | WB |
| p-IκBα | TP56280 | Abmart | 1:1000 | WB |
| IκBα | T55026 | Abmart | 1:1000 | WB |
| β-actin | 20536-1-AP | Proteintech | 1:1000 | WB |
| Anti-rabbit IgG-HRP | 7074 | Cell signalling technology | 1:5000 | WB |
| Anti-goat IgG-HRP | RS2123 | Immunoway | 1:5000 | WB |
| α-SMA | ab124964 | Abcam | 1:200 | Immunofluorescence |
| fibronectin | ab268020 | Abcam | 1:50 | Immunofluorescence |
| Desmin | WL0174 | Wanleibio | 1:100 | Immunofluorescence |
| CD31 | AF3628 | R&D Systems | 1:200 | Immunofluorescence |
| F4/80 | HA721520 | HUABIO | 1:200 | Immunofluorescence |
| COL1 | 1310-01 | SouthernBiotech | 1:100 | Immunofluorescence |
| Ly6G (FITC Conjugate) | 88876 | Cell signalling technology | 1:100 | Immunofluorescence |
| Goat Anti-Rabbit IgG H&L (Alexa Fluor 488) | ab150077 | Abcam | 1:500 | Immunofluorescence |
| Goat Anti-Rabbit IgG H&L (Alexa Fluor 594) | ab150080 | Abcam | 1:500 | Immunofluorescence |
| Donkey Anti-Goat IgG H&L (Alexa Fluor 594) | ab150132 | Abcam | 1:500 | Immunofluorescence |
| Donkey Anti-Goat IgG H&L (Alexa Fluor 488) | ab150129 | Abcam | 1:500 | Immunofluorescence |

**Supplementary Table 3.** Sequences of shRNAs.

| **Species** | **Gene** | **Sequences** |
| --- | --- | --- |
| Human | RUNX1-1 | CCTACGATCAGTCCTACCAAT |
| Human | RUNX1-2 | CCTCGAAGACATCGGCAGAAA |
| Human | ITGB1-1 | GCCTTGCATTACTGCTGATAT |
| Human | ITGB1-2 | CCAAATCATGTGGAGAATGTA |
| Murine | RUNX1-1 | CACCTACCATAGAGCCATCAA |
| Murine | RUNX1-2 | GCCCTCCTACCATCTATACTA |
| Murine | ITGB1-1 | GCACGATGTGATGATTTAGAA |
| Murine | ITGB1-2 | CCCGACATCATCCCAATTGTA |

**Supplementary Table 4.** Clinicopathologic characteristics of PDAC patients in the Chongqing RNA-seq cohort.

| Characteristics | PDAC NLM, n(%)  (n=10) | PDAC LM, n(%)  (n=6) | *P* value |
| --- | --- | --- | --- |
| **Age (years), median (range)** | 60 (42-71) | 61.5 (56-73) | 0.635 |
| **Gender** |  |  |  |
| Male | 7 (70.0) | 2 (33.3) | 0.302 |
| Female | 3 (30.0) | 4 (66.7) |  |
| **Tumor** **Differentiation** |  |  |  |
| Moderately-differentiated | 6 (60.0) | 1 (16.7) | 0.145 |
| Poorly-differentiated | 4 (40.0) | 5 (83.3) |  |
| **Tumor Location** |  |  |  |
| Pancreatic head | 8 (80.0) | 3 (50.0) | 0.299 |
| Pancreatic body/tail | 2 (20.0) | 3 (50.0) |  |
| **Tumor size** | 3.0 (2.0-5.5) | 4.1 (3.8-5.0) | **0.016** |
| **T stage** |  |  |  |
| T1-T2 | 9 (90.0) | 3 (50.0) | 0.118 |
| T3-T4 | 1 (10.0) | 3 (50.0) |  |
| **Smoking status** |  |  |  |
| Present or former smoker | 1 (10.0) | 2 (33.3) | 0.518 |
| Nonsmoker | 9 (90.0) | 4 (66.7) |  |
| **Alcohol Drinking History** |  |  |  |
| Yes | 2 (20.0) | 3 (50.0) | 0.299 |
| No | 8 (80.0) | 3 (50.0) |  |
| **Hepatitis/Cirrhosis History** |  |  |  |
| No | 10 (100) | 6 (100) | - |
| **Preoperative Neoadjuvant Therapy** |  |  |  |
| No | 10 (100) | 6 (100) | - |

Continuous variables are presented as median (range) and compared using the Mann–Whitney U test. Categorical variables are presented as n (%) and compared using Fisher’s exact test.

**Supplementary Table 5.** Particle concentration, protein concentration, and particle-to-protein ratios of EV preparations used in this study.

| EVs |  | Particle concentration  (particles/mL) | Protein concentration  (μg/mL) | Particle/Protein  (particles/μg) |
| --- | --- | --- | --- | --- |
| CFPAC-1 shNC | #1 | 3.67 × 10^11^ | 116.72 | 3.14 × 10^9^ |
|  | #2 | 3.92 × 10^11^ | 119.16 | 3.29 × 10^9^ |
|  | #3 | 3.13 × 10^11^ | 146.08 | 2.14 × 10^9^ |
| CFPAC-1 shRUNX1 | #1 | 3.29 × 10^11^ | 80.21 | 4.10 × 10^9^ |
|  | #2 | 3.81 × 10^11^ | 151.66 | 2.51 × 10^9^ |
|  | #3 | 4.14 × 10^11^ | 126.36 | 3.27 × 10^9^ |
| Pan02 shNC | #1 | 4.32 × 10^11^ | 224.39 | 1.93 × 10^9^ |
|  | #2 | 4.72 × 10^11^ | 296.70 | 1.59 × 10^9^ |
|  | #3 | 5.12 × 10^11^ | 243.52 | 2.10 × 10^9^ |
| Pan02 shITGB1 | #1 | 4.07 × 10^11^ | 179.55 | 2.27 × 10^9^ |
|  | #2 | 4.60 × 10^11^ | 263.65 | 1.74 × 10^9^ |
|  | #3 | 6.05 × 10^11^ | 305.79 | 1.98 × 10^9^ |
| CFPAC-1 shNC | #1 | 6.84 × 10^11^ | 651.20 | 1.05 × 10^9^ |
|  | #2 | 3.06 × 10^11^ | 181.99 | 1.68 × 10^9^ |
|  | #3 | 6.32 × 10^11^ | 231.70 | 2.73 × 10^9^ |
| CFPAC-1 shITGB1 | #1 | 5.04 × 10^11^ | 206.10 | 2.45 × 10^9^ |
|  | #2 | 4.76 × 10^11^ | 224.75 | 2.12 × 10^9^ |
|  | #3 | 6.67 × 10^11^ | 517.53 | 1.29 × 10^9^ |

Values from three independent EV preparations per group are listed individually.
